# Regulation of differentiation and generation of osteoclasts in rheumatoid arthritis

**DOI:** 10.3389/fimmu.2022.1034050

**Published:** 2022-11-18

**Authors:** Qing Niu, Jinfang Gao, Lei Wang, Jiaxi Liu, Liyun Zhang

**Affiliations:** ^1^ School of Basic Medical Sciences, Shanxi Medical University, Taiyuan, China; ^2^ Shanxi Bethune Hospital, Shanxi Academy of Medical Sciences, Tongji Shanxi Hospital, Third Hospital of Shanxi Medical University, Taiyuan, China

**Keywords:** osteoclasts, rheumatoid arthritis, bone destruction, regulation of formation and differentiation, targeted therapy

## Abstract

**Introduction:**

Rheumatoid arthritis (RA), which affects nearly 1% of the world’s population, is a debilitating autoimmune disease. Bone erosion caused by periarticular osteopenia and synovial pannus formation is the most destructive pathological changes of RA, also leads to joint deformity and loss of function,and ultimately affects the quality of life of patients. Osteoclasts (OCs) are the only known bone resorption cells and their abnormal differentiation and production play an important role in the occurrence and development of RA bone destruction; this remains the main culprit behind RA.

**Method:**

Based on the latest published literature and research progress at home and abroad, this paper reviews the abnormal regulation mechanism of OC generation and differentiation in RA and the possible targeted therapy.

**Result:**

OC-mediated bone destruction is achieved through the regulation of a variety of cytokines and cell-to-cell interactions, including gene transcription, epigenetics and environmental factors. At present, most methods for the treatment of RA are based on the regulation of inflammation, the inhibition of bone injury and joint deformities remains unexplored.

**Discussion:**

This article will review the mechanism of abnormal differentiation of OC in RA, and summarise the current treatment oftargeting cytokines in the process of OC generation and differentiation to reduce bone destruction in patients with RA, which isexpected to become a valuable treatment choice to inhibit bone destruction in RA.

## Differentiation and function of OCs under physiological condition

OCs are derived from monocytes in the monocyte-macrophage lineage (1) and can phagocytise foreign particles, produce cytokines and act as antigen-presenting cells (2). The identification criteria demonstrated by Ziegler-Heitbrock et al. are considered the most appropriate current classification (3). This criterion classifies human monocytes into three subpopulations: classical monocyte (CD14++CD16−), intermediate monocyte (CD14++CD16+), and non-classical monocyte (CD14+CD16++). Komano et al. (4) believed that classical monocyte subsets (CD14++CD16 -) are circulating OC precursors (OCP) that can differentiate into OCs. OC production is a multi-step process. In the early stage, hematopoietic stem cells (HSC) proliferate in the macrophage line; in the middle stage, CTR and tartrate-resistant acid phosphatase (TRAP) enter and express OCP; and late OCP fuse and become mature multinucleated cells. Then OCs began to play the role of osteolysis (5). OCs differentiation mainly depends on macrophage colony stimulating factor (M-CSF) and nuclear factor κB receptor activator ligand (RANKL) signals, which mediate all epigenetic and transcriptional changes of OCs (6). Local environmental factors also contribute to establishing tissue-specific transcriptional profiles in monocyte subsets (7, 8).

**Figure 1:**
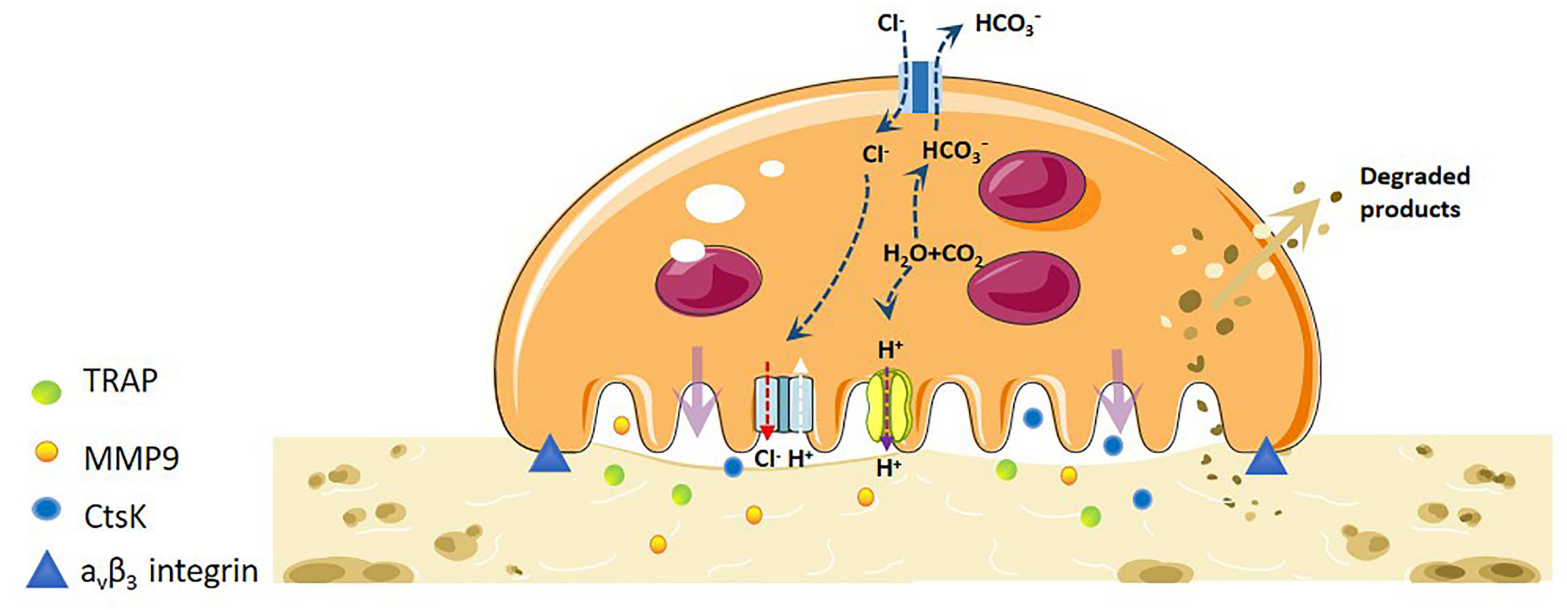
Mechanistic differentiation and function of OC under physiological conditions. OCs are the only osteolytic cells *in vivo*. It dissolves bone matrix in four steps. The first step is OC adhesion. The integrin and actin microfilaments expressed by OCs allows the cells to closely adhere to the bone surface and form a special cytoskeleton. The second step is OC polarisation, and the OC plasma membrane and cytoskeleton are recombined. The plasma membrane of OCs is divided into four different regions: basal region, wrinkle edge, sealing region and functional secretory region. The third step is degradation of bone matrix. H-ATPase transports H+ to the cavity, allowing the pH of the cavity microenvironment to near a pH of 4-6. This acidic environment loosens the minerals in the bone matrix, and the wrinkle edge also secretes protons and various bone resorption enzymes to degrade organic matter. The fourth step is the removal of degraded products. The degraded bone matrix products are transported from the fold edge to the functional secretory region through endocytosis, and then released extracellularly.

Mature OCs are a 20-100tzm in diameter, containing 2-20 nuclei, cytoplasm rich in mitochondria, lysosomes, ribosomes, Golgi bodies and other organelles, pseudopodia and processes of irregular cells, is the only cell in the body with the ability to dissolve bone tissue (9). The integrin (AVβ3-integrin) and actin microfilament (actin) expressed on OCs interact with extracellular MMP, which makes the cells adhere closely to the bone surface, and OCs begin to adsorb (10). After the completion of the adsorption process, the plasma membrane and cytoskeleton of OCs were reorganised. During the recombination process, the plasma membrane of OCs was divided into four different regions: basal region, fold margin, sealing area and functional secretory region (11, 12). In order to increase the efficiency of bone resorption, the cell membrane facing the bone surface is folded to form a larger bone resorption space-fold margin. The formation of a wrinkle margin enables it to widely seal the bone surface and improve the efficiency of bone resorption. In the fold margin, OCs open Cl- channels (H+Cl-exchange transporters) and secretes vacuolar H+-ATP enzymes, which acidify the extracellular space, making the pH of the lacunar microenvironment close to 4-6. This acidic environment can dissolve calcium from bone, loosen minerals in the bone matrix, and create an optimal environment for creatine kinase (CK) to enter the lacunar hydrolysed organic matrix(such as bone type I collagen) (13). Concurrently, the fold margin secretes protons and various bone resorption enzymes such as TRAP, MMPs and cathepsin K (CtsK). The inorganic components of bone are degraded by H+, Cl- and TRAP, while the organic components are degraded by various enzymes such as CtsK and MMP-9 (14). The fold is maintained by a sealed area regulated by αvβ3-integrin, which promotes the directed secretion of enzymes from the outer space of cells to this region (15) (**Figure 1**).

## Pathogenic mechanisms of OCs in bone destruction in RA

In 1998, Gravallese et al. (16) used immunohistochemistry and molecular techniques to find that OCPs gathered at the junction of synovial pannus, which proved that OCs played a key role in the process of RA bone erosion. RA bone destruction is the pathological result of the interaction between bone and immunity. The interaction between cells and inflammatory factors leads to changes in joint microenvironment and affects the differentiation of OCs.

Now it has been found that a large number of pro-inflammatory cytokines affect OC. The cytokines that promote the differentiation or activation of OC are IL-1, IL-6, IL-8, IL-11, IL-17, TNF- α, Th17 cells and so on. The cytokines that inhibit the differentiation or activation of OC are IL-4, IL-10, IL-13, IL-18, IFN- γ, IFN- β, IL-7, IL-12, IL-23. IL-6 and TGF- β have dual effects, and their net effects depend on the developmental stage of OC (17).

These cytokines mediate the regulation of bone destruction by OC through multiple signal pathways, mainly RANK/RANKL/OPG signal pathway (18). When RANK binds to RANKL, the tumor necrosis factor receptor related factor (TRAF) family is recruited and activates downstream signaling pathways, including NF- kappa B, MAPKs and AP-1 (19), which eventually activate the nuclear factor of activated T cell C1 (NFATc1), which is the main transcription factor of OC differentiation. RANKL regulates the activation of NFATcl and promotes the differentiation of OC through two signal pathways: NF- κ B/AP-1/c-fos and calcium signal pathway (20). Osteoprotegerin (OPG) is a decoy receptor that competes with RANKL to down-regulate the differentiation and production of OC with binding RANK. Wnt signaling pathway up-regulates the expression of OPG and plays an anti-OC role, while TNF- α or RANKL regulates the expression of c-Fos and NFAT-c through CREB phosphorylation to induce osteoclast differentiation (21).

Deeply understands the differentiation and formation process of OC and the mechanism of bone resorption, and reduces the conditions for promoting OC activation, which can provide new ideas for the treatment of early RA, prevent disability and reduce mortality in RA patients.

## Regulation of differentiation and generation of OCs in RA

The maintenance of normal bone mass is the result of the joint action of osteoblasts(OBs) and OCs. Bone formation promoted by OBs is balanced with bone resorption caused by OCs so that bone mass can be maintained in a relatively stable state. The imbalance between the two is the main cause of systemic bone loss and joint local bone destruction in patients with RA.

The abnormal differentiation of OCs will have a significant impact on the progress of bone destruction in RA. This process is strictly regulated by genes and epigenetic determinants, which will play an important role in both transcription and post-transcription. In addition, the *in vitro* environment is also a key factor that can not be ignored in the development and progression of RA. We will discuss these factors that affect OCs differentiation as follows.

### Differentiation regulation of OCs

#### Macrophage-stimulating factor

M-CSF is an essential cytokine that promotes the differentiation and maturation of OCPs and induces OC formation by binding to the cell surface receptor—C-FMS (22). M-CSF induces the expression of dendritic cells specific transmembrane protein (DC-STMAP) and nuclear factor of activated T cells 1(NFATc1), which then leads to the fusion of OCPs *in vitro* bone marrow-derived cells, resulting in the formation of multinucleated giant cells (23). In addition, M-CSF can induce the expression of RANK in bone marrow cells, which can interact with RANKL and induce the differentiation of OCs (24). M-CSF plays a dual role in OCs genesis. It can promote or inhibit OC differentiation, depending on the type and pathway of OCPs. In inflammatory arthritis, there is a different group of OCPs, which can respond to a variety of pro-inflammatory cytokines to compensate for the impact of the loss of the CSF-1 signal pathway on OC differentiation (25, 26).

#### Toll-like receptors

Toll-like receptors (TLR) are important innate immune receptors. Some studies have shown that the expression of TLR in joints of patients with RA is increased. TLR3 and TLR4 were highly expressed in the early stage of RA (27), and TLR2 was highly expressed in cartilage and bone erosion sites (28).

OCs can also express TLRs, and its expression pattern varies with the stage of OCs. OCPs express TLR1-TLR9, but in the process of their differentiation into mature OCs, only TLR2 and TLR4 are mainly expressed (29). It is suggested that these two TLR play an important role in OC genesis. Lu et al. (30) have shown that patchoulol can inhibit OC activity and bone resorption by regulating the expression of TLR4.

In the clinical model of RA, the positive effects of TLR2,4,5 and 7 have been confirmed. Intra-articular injection of a TLR2 or TLR4 ligand can induce joint inflammation and chronic destructive arthritis (31, 32). Local injection of TLR5 ligands can enhance joint inflammation and bone destruction in the collagen-induced arthritis (CIA) model (33). Moreover, in the CIA arthritis model, the absence of TLR7 reduced joint inflammation and arthritis-induced bone loss (34, 35). Kim et al. (36) studied the effect of TLR7 on RANKL expression and OC generation and found that TLR7 and RANKL co-located in RA-FLS, TLR7 can directly regulate OC differentiation, and stimulate synovial fibroblasts (FLS) to produce RANKL. This study provides the first evidence that TLR7 pathway is involved in RA bone destruction. Further exploration of the specific mechanisms of other TLR involvement in OCs is needed.

#### Tyrosine kinase 3

Tyrosine kinase 3 (Tyro3TK) is a member of the receptor tyrosine kinase family involved in immune homeostasis (37). It can be expressed on the cell membrane of monocytes/macrophages, dendritic cells, natural killer (NK) cells and nerve cells (38). In 1998, Nakamura et al. (39) first found that Tyro3TK can be expressed in multinucleated OCs. When it binds to a mature ligand–Gas6, the bone resorption activity of mature OCs is enhanced, while Tyro3TK does not affect the differentiation of OCs and bone marrow cells. Katagiri et al. (40) also found that Tyro3TK can be detected in mature OCs. Gas6 is widely expressed in osteocytes and stimulates the function of OCs (41). Ruiz-Heiland (42) showed that in an arthritis model, Tyro3TK deficient mice showed increased bone mass and impaired OC differentiation, indicating that Tyro3TK was involved in the differentiation and functional maturation of OCs.

Komano et al. (4) believed that CD14+CD16- monocytes are the main precursors of RA-OCs. The expression of Tyro3TK is enriched in CD14+CD16-monocytes and up-regulated in RA patients, and is positively correlated with disease manifestations such as IgM level, tenderness joint count and disease activity score (43). Gas6 is the ligand of Tyro3TK, and the combination of the two can regulate the clearance of apoptotic cells, cytokine production, cell proliferation, thrombosis and hematopoiesis (44). Gas6 is expressed in synovial tissue and cell fluid in RA and plays a role in the survival of RA synovial endothelial cells (45). The evaluation of Gas6 in synovial tissue and synovial fluid of RA found that Gas6 can promote synovial proliferation of RA and is related to bone destruction of RA (46). Gas6 can promote the OC formation of CD14+ CD16− monocytes while disrupting the interaction between Gas6 and Tyro3TK. The number of OCs differentiated from CD14+ CD16− monocytes decreased significantly with a dose-dependent anti-Tyro3TK antibody (43). Unlike Tyro3TK, two other members of the TAM family, Ax1 and Mer, play a protective role in RA. Mer reduces the release of pro-inflammatory molecules such as tumour necrosis factor (TNF) and IL-6 by binding to Gas6 and induces anti-inflammatory mediators such as IL-10 (47). *In vitro* studies have confirmed that anti-TNF drugs can inhibit pro-inflammatory cytokines, up-regulate IL-10, activate the positive feedback mechanism involving the Gas6/Mer axis, and limit the inflammatory cascade reaction (48). Concurrently, activated T cells enhance the inhibitory ability of Treg through Axl/Gas6 and enhance the anti-inflammatory ability of Gas6 again (49). When joint inflammation decreases, OC generation also decreases.

These results suggest that the up-regulation of Tyro3TK expression plays a key role in OC genesis and bone destruction in inflammatory arthritis. However, the detailed signal mechanism of Tyro3TK on RA-CD14+CD16- still needs further research.

#### ACPA

Epidemiological evidence shows that bone loss is closely associated with positive anti-citrullinated protein antibody (ACPA) in patients with RA (50–52). Higher titers of ACPA are associated with systemic bone loss, suggesting that ACPA may lead to bone loss either directly or indirectly by increased systemic inflammation. Similar to macrophages, OCPs express FCγR (53–55), in which FcγRI, FCγRIIB and FCγRIIIA are significantly up-regulated during OC formation *in vitro (* 54). In the mouse model of inflammatory arthritis, the specific deletion of OCs FcγRIV resulted in abnormal OC formation and osteoprotective effects in inflammatory joints, and activation of FcγR with cross-linked antibodies could promote OC differentiation of mouse bone marrow cells (55), suggesting that FcγR has a regulatory effect on OC activity and bone resorption.In addition, low Fc sialylation of ACPA contributes to maintaining the OC phenotype.

It is well known that IL-8 (56) is the key medium for ACPA-induced OC activation. Krishnamurthy et al. (57) showed that serum and joint-derived ACPA can promote OC generation by affecting the secretion of IL-8 by OCs themselves.

The emergence of autoantibodies is a unique characterization of autoimmune diseases such as rheumatoid arthritis. The evidence that ACPA is related to osteoclast differentiation provides sufficient evidence for bone immunology. In addition to ACPA, people can also extensively explore the effects and specific mechanisms of other autoimmune antibodies on osteoclasts. I think this can start from clinical research.The emergence of autoantibodies is a unique characterisation of autoimmune diseases such as RA, and the evidence that ACPA is associated with OC differentiation provides sufficient evidence for osteoimmunology. In addition to ACPA, clinical research can extensively explore the effects of other autoimmune antibodies on OCs and the specific mechanism of action.

#### B cell

B cells bind to soluble APRY produced by TNF superfamily B cell activating factor (BAFF) (also known as BLyS) and peripheral blood mononuclear cells (PBMC) to promote B cell proliferation. Autoreactive B cells differentiate into plasma cells, producing RF and ACPA, which aggravate synovitis by forming immune complexes and activating complements, especially in the early stages of RA (58, 59). Fcγ RIIB is an important regulator of B cells, and its deletion will lead to the induction of many autoantibodies (60), thus promoting the formation of OCs. B cells also produce TNF-α.

The role of RANKL expressed by lymphocytes in various types of bone injury has not been elucidated. In the bone marrow of arthritic mice, Komatsu et al. (61) found that the number of plasma cells expressing RANKL increased significantly, and the cells had the ability to induce OC formation *in vitro*. The genetic ablation of RANKL in B lineage cells can reduce periarticular bone loss in autoimmune arthritis mice. Specific to RA, it was recently shown that CD27+IgD-switched memory B-cells produce RANKL in quantities exceeding that produced by T-cells upon stimulation, and synovial RA B cells spontaneously produce RANKL and promote greater osteoclastogenesis than B cells from healthy controls (62).

#### T cell

Bone damage caused by immune system abnormalities has been a challenging problem for a long time. The main cells involved in the pathological progression of RA are CD4+T cells, which are related to subsequent bone destruction (63), but which kind of CD4+T cells induce OC generation and the mechanism are not fully understood.

CD4+T cells can differentiate into Th1, Th2, Th17 and Treg subsets (64). RANKL is expressed in activated T cells, which can directly act on OCPs to induce OC differentiation. The interaction of inducible costimulatory molecules (ICOS) expressed by activated T cells with ICOS ligand (B7h or CD275) expressed by OC-like cells (MDOCs) derived from monocytes can block their differentiation into OCs. This interaction down-regulates the expression of TRAP, NFATc1 and OSCAR during the maturation of MDOCs to OCs (65).

Among them, Th1 and Th2 cells inhibit the occurrence of OCs mainly through the action of IFN- γ and IL-4 on progenitor cells (66). Previous studies have shown that both IL-12 and IL-18, which drive Th1 differentiation, inhibit OC generation through IFN- γ or GM-CSF (67), while IL-10 released by Th2 cells also negatively regulates OC generation (68), which further supports the negative effects of Th1 and Th2 cells on OC formation.

Th17 cells are a new subgroup of CD4+T cells, which mainly secrete cytokines such as IL-17A, IL-17F and IL-21, and overexpress transcription factors retinoic acid-related orphan nuclear receptors (RORγ) and ROR α, which play an important role in autoimmune diseases. Satok et al. (66) found that Th17 cells promote OC generation by producing IL-17. On the one hand, IL-17 indirectly promotes OC generation by inducing RANKL expression through the IL-23-IL-17 axis. Conversely, IL-17 releases a large number of inflammatory cytokines, such as TNF-α and IL-1 (69, 70), by recruiting and activating immune cells. These inflammatory cytokines promote the expression of RANKL in cells related to OC generation and activate OCPs in cooperation with RANKL signalling pathway to affect OC generation. Th17 cells are called OC generation-related Th subsets, not only because Th17 cells have a positive effect on OC genesis *in vitro* but also because they shift joint microenvironment balance in a direction conducive to OC differentiation *in vivo*. Th17 cells stimulate RANKL expression, RANKL interacts well with CD14+ monocytes, and CD14+ monocytes expressed CCR6 (71) (the surface marker of Th17 cells). This suggests that Th17 cells play a key role in OC differentiation. Th17 cells can also mediate synovial neovascularisation, leading to bone erosion (72).

Treg cells exert an immune effect by secreting IL-10 and TGF- β to maintain lymphocyte homeostasis and tolerance and reduce the production of inflammatory cytokines and antibody secretion. Th17 cells and Treg cells coordinate *in vivo (* 73). When the number of Th17 cells increases, hyperfunction, the number of Treg cells decreases, low function, resulting in an imbalance of Th17/Treg, synovitis, joint destruction, bone erosion and other manifestations, aggravating the development of the course of RA.

#### Macrophages

Myeloid cells play an important role in synovial inflammation, and the abundance of synovial macrophages is related to the degree of joint erosion (74). Firstly, macrophages are important cells producing cytokines in the joints of patients with RA, the main sources of TNF-α and IL-6, as well as other cytokines and chemokines involved in the disease process, such as IL-1β, IL-8 and chemokine C motif ligand 2 (CCL-2) (75). Secondly: circulating monocytes and resident macrophages are the precursors of OCs. These monocytes are continuously supplied to the inflammatory synovium and differentiate into OCs in RA.

According to the expression of surface molecules, macrophages are divided into pro-inflammatory (M1) and anti-inflammatory (M2) phenotypes (76). Synovial macrophages from patients with RA have M1 phenotype, highly express proteins such as PHD3, CCR2, MMP-12 and TNF-α (77). In RA, high activation of macrophages increases the expression of TLR2, TLR3, TLR4 and TLR7, causes NF-κB to activate and inhibit the expression of RANK, attenuates the transmission of RANKL and CSF-1 signaling pathways (78) and promotes synovitis and cartilage destruction by producing enzymes, cytokine and other inflammatory factors. Contrastingly, activated macrophages significantly increase the level of synuclein A (activin A) encoded by INHBA gene, which mediated M1 polarization. Polarized M1 macrophages secrete many pro-inflammatory cytokine (IFN-γ, TNF-α, IL-1 and IL-6), chemoknockoutines (CCL5, CXCL-1 and CXCL-10) and various matrix lyases, which in turn activate fibroblasts and Ocs. Furthermore, this helps recruit neutrophils, monocytes, and lymphocytes, and triggers a series of inflammatory reactions that accelerate inflammation and lead to destruction of articular cartilage (79, 80).

Further, macrophage autophagy also plays an important role in the pathogenesis of RA, the expression of autophagy signal pathway members (ATG7 and Beclin-1) in RA-Ocs is up-regulated, and myeloid cell-specific Atg7 deletion occurs not only in Ocs, but also in monocytes (81); autophagy of monocyte-derived macrophages in RA joints may also be involved, thus helping enhance the bone resorption activity.

#### Fibroblasts

**Figure 2:**
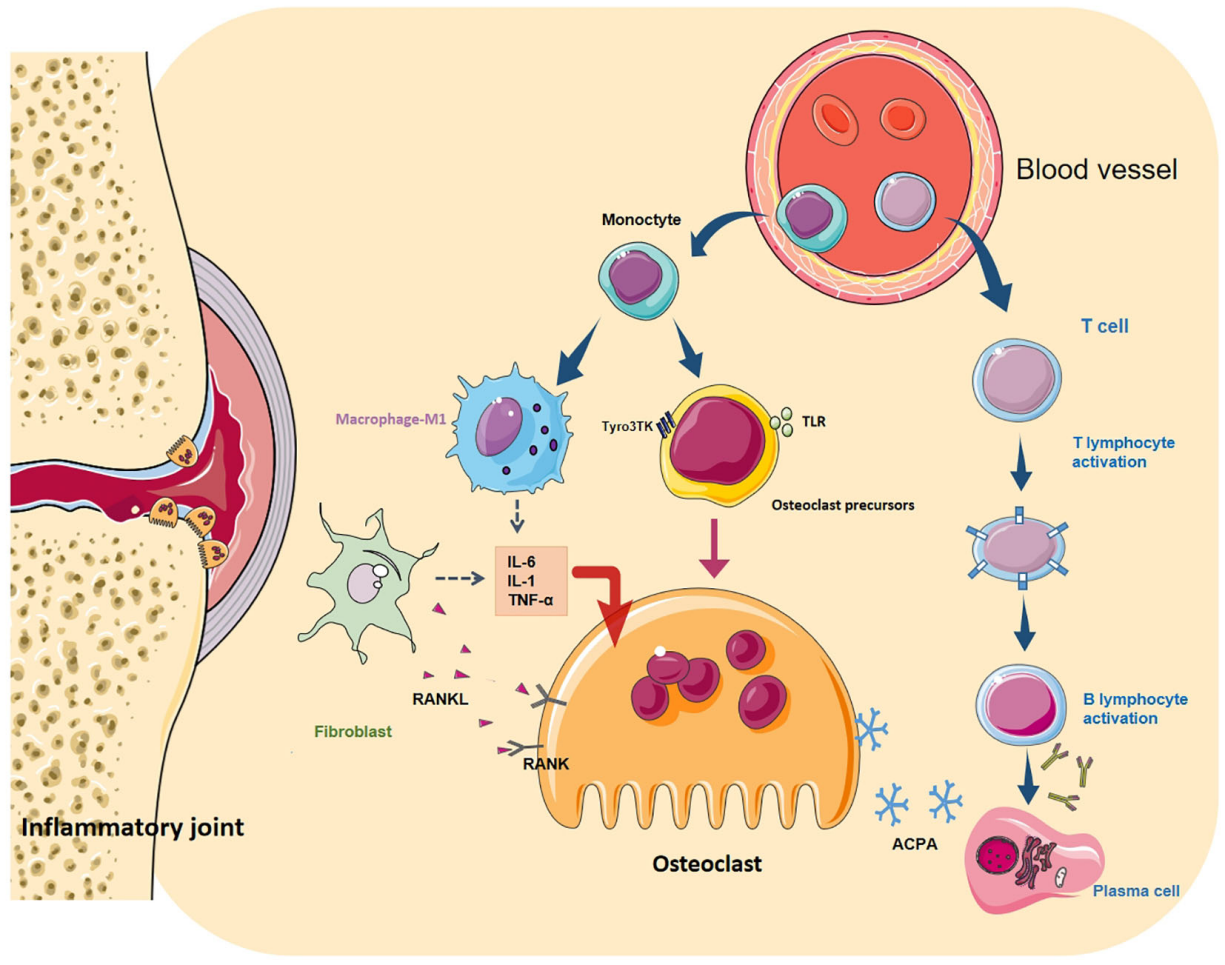
Differentiation regulation of osteoclasts. OC differentiation is a multi-step process, in which cytokines and various cell interactions affect the differentiation of OC from the mononuclear to the terminal state. Among them, autoantibodies directly act on OCs (and their precursors) or cooperate with T and B cells to form a vicious circle. Toll-like receptors (TLR) and Tyro3TK on the surface of OCPs bind to their specific ligands to affect OC differentiation. M1 macrophages secrete many pro-inflammatory cytokines, chemokines and various matrix lyases to activate OCs and aggravate the inflammatory reaction. FLS is the key cell in RA bone destruction. Various pro-inflammatory factors allow it to express RANKL in large quantities. It binds to RANK on the surface of macrophages and OCs and promotes the maturation of OCs through NF- κB signal cascade.

Fibroblasts (FLS) are the core cell in the pathogenesis of RA, and their tumour-like abnormal proliferation is the main cause of synovial hyperplasia (82). High levels of pro-inflammatory cytokine, chemokines and matrix metalloproteinases (MMP) secreted by FLS during the disease to mediate inflammation, bone erosion, absorption, and cartilage degradation, and finally lead to the destruction of bones and joints. One key factor in transforming FLS into an invasive RA-FLS type is IL-17. IL-17 can directly act on synovial fibroblasts to stimulate the release of MMP to decompose cartilage. Concurrently, IL-17 cooperates with IL-1 and TNF-α to induce the secretion of pro-inflammatory cytokine and chemokines, affect the metabolic pathway of monocytes and synovial fibroblasts, promote synovial cells to have an inflammatory response, and directly assist FLS-mediated RA progression (46). Conversely, RANKL expressed by synovial fibroblasts is mainly responsible for the formation of OC and bone erosion in inflammatory arthritis (83). It binds to the receptor (RANK) on the surface of activated OCs and initiates the IKK/NIK pathway through TRAF2, 5 and 6 proteins, leading to the activation of downstream NF-κB, MAPK, NFATc1 and Src signal cascades (84). Activation of these transcription factors can stimulate the expression of TRAP, CtsK and MMP-9 genes in the nucleus, promoting OC generation and bone resorption (**Figure 2**).

### Generation regulation of OCs

#### DNA methylation

DNA methylation affects the differentiation of OCs and bone resorption. Nishikawa et al. (85) found that DNA methyltransferase (DNMT) 3a promotes OC differentiation and bone resorption by inhibiting interferon regulatory factor-8(IRF8), a negative regulatory factor associated with OC differentiation, DNMT3a inhibits IRF8 by increasing the methylation of IRF8 distal regulatory elements and increase the concentration of S-Adenosylmethionine (SAM) to promote DNA methylation.

Moreover, DNMT3a and DNMT3b interact with PU.1, an important OC transcription factor, and form complexes through DNA methylation to regulate the target gene of PU.1 (86). The expression of RANKL and osteoprotegerin (OPG), the key factors regulating OC, is closely related to the methylation of its promoter region. After demethylation treatment, mice can significantly increase the expression of RANKL and OPG, thus affecting the production of OCs (87).

#### Histone acetylation

The N-terminal of histone can undergo various covalent modifications, such as acetylation, methylation, phosphorylation, and ubiquitin (88). Histone acetylation usually promotes gene transcription, while deacetylation inhibits gene transcription. Kim et al. (89) found that RANKL can promote the acetylation of NFATc1 in OCs through acetyltransferase (HAT), and then promote its expression and protein stability, while HADc5 overexpression reduces the acetylation of NFATc1, and the use of HDAc inhibitor sodium butyrate can promote the acetylation of NFATc1, and the subsequent differentiation of OCs. Yasui et al. (86) used ChIP sequencing technology to prove that the histone precursor H3 lysine 4 (H3K4me3) exists in the NFATc1 gene of OCPs, but significantly decreased in mature OCs. Jumonji domain-containing protein3(JMJD3), an H3K27 demethylase, inhibits OC differentiation induced by RANKL by inhibiting H3K27 methylation in the promoter region of NFATc1.

#### microRNA

MicroRNA is a 19-22 BP non-coding RNA that can regulate gene expression after transcription (90). In the process of differentiation from OCP to mature OCs, the expression of 44 kinds of miRNA increased more than twice (91).

Among them, miR-218, miR-503 and miR-125 inhibited the differentiation of OCs. During the differentiation of BMMs and RAW264.7 cells into OCs induced by RANKL, the expression of miR-218 decreased significantly, and the overexpression of miR-218 significantly inhibited the differentiation of OCs (92). Through bioinformatics analysis, Chen et al. (93) found that RANK is the direct target of miR503. The binding of miR-503-5p to the 3’-UTR region of RNAK mRNA inhibits the translation of RANK protein, thus inhibiting OC differentiation. Guo et al. (94) showed that miR-125a down-regulated the expression of TRAF6, and then significantly decreased the expression of NFATc1 to inhibit OC differentiation.

However, miR-214, miR-31a and miR-223 promote the differentiation of OCs. Zhao et al. (95) found that miR-214 significantly increased the expression of miR-214 during the differentiation of BMMs into OCs induced by M-CSF and RANKL by regulating the PTEN/P13k/Akt signal pathway, which promoted the differentiation of OCs. The expression of miR-31 is reportedly significantly increased in OC differentiation induced by RANKL, and OC differentiation is significantly inhibited after antagonising the function of miR-31 (96) MiR-223 is an important regulator of myeloid cell differentiation, which is significantly up-regulated in neutrophils and macrophages (97) and participates in OC generation. As an important regulator of OC differentiation, IKK- α is the proven target gene of miR-223 (98), contributing to RA development. The up-regulation of miR-223 expression suppresses the expression of OC target nuclear factor 1A (NF-1A) (3) and affects the generation of OC. According to the study conducted by Li. et al. (99), which compared with the OA group, the expression of miR-223 in the synovium of RA patients and CIA mice increased. A single intraperitoneal administration of miR-223T lentiviral vector decreased arthritis and histological score and miR-223 expression and inhibited OC generation and bone erosion in the joints of CIA mice.

#### Glycosylation

Glycosylation regulates the localization, function and activity of proteins in tissues and cells, and affects a variety of important life activities, such as cell recognition, differentiation, signal transduction, immune response and so on (100). Protein glycosylation can be divided into different modification types, among which N-glycosylation and O-GlcNAc glycosylation are the two main modification types. O-GlcNAc glycosylation can regulate inflammation, and its dynamic changes are necessary for osteoclast genesis (101, 102). Li-Yi (103) found that in the early stage of OC, increasing O-GlcNacylation can promote OC differentiation through oxidative phosphorylation and intercellular fusion, while it will be down-regulated in mature stage. TNF-α can also promote the dynamic regulation of O-GlcN acylation to increase osteoclast formation in inflammatory arthritis.Ttargeted drugs or gene inhibition of O-GlcNAc transferase (OGT) or O-GlcNAcase (OGA) could prevent osteoclast differentiation and improve bone loss in the early and late stage of differentiation, respectively. O-GlcNacylation derived from hexosamine biosynthesis pathway is very important for osteoclast differentiation mediated by RANKL (104). Both N-acetylglucosamine (GlcNAc) and glucosamine can inhibit osteoclast differentiation through the Modulation of Glycosylation Including O-GlcNAcylation (105). We already know that antibody glycosylation inhibits the differentiation and maturation of osteoclasts (54). The Fc segment of IgG can be glycosylated by different sugar segments (106), and the increase of IgG glycosylation can alleviate the symptoms and progress of RA (107). Estrogen can protect joints from arthritis by increasing immunoglobulin glycosylation and inhibiting osteoclast activation (108).Phytoestrogens can bind to estrogen receptor (ER) and induce estrogen-like activity (109), which negatively regulates the differentiation of RAW264.7 cells into osteoclasts (110). Ning et al. found that phytoestrogens can protect mice from CIA by increasing immunoglobulin glycosylation, reducing inflammation, inhibiting NFATc1/c-Fos and reducing osteoclast activity (111).At present, glycosylation is the focus of scientific research. Any minor modification of glycosylation may affect the location and stability of cell surface receptors and their sensitivity to signal molecules, and affect the function of osteoclasts. The regulation of its effect on osteoclast formation and differentiation should be further explored.

#### Ubiquitination

Ubiquitination modifications are one of the main pathways of protein degradation. Ubiquitinylated and deubiquitinylated enzymes ensure the stability and proper function of most cellular proteins. The ubiquitin-proteasome system plays an important role in the regulation of bone reconstruction, and ubiquitin-specific proteases (USPs) are a major component of this system (112). USP26 stabilizes β-catenin to impairs the osteoclastic differentiation of bone myelomonocytes (BMMs) by stabilizing inhibitors of NF-κBα (IκBα) (113).Conditional knockdown of USP34 in bone marrow-derived macrophages (BMMS) or osteoclasts, USP34 inhibits NF-κB signaling by deubiquitinating and stabilizing the NF-κB inhibitor alpha (IκBα),leading to enhanced osteoclast function and reduced bone mass (114). Cylindromatosis (CYLD), a USP, reduces ubiquitination levels of TRAF6 protein and inhibits the RANK signalling pathway, which is essential for osteoclast differentiation (115). In addition to this, Protein Tyrosine Phosphatase Receptor Type JPTPRJ reduced the ubiquitination and degradation of NFATc1, a key osteoclast transcription factor, thereby inhibiting OC maturation (116). (+)-Vitisin A inhibits osteoclast differentiation by blocking TRAF6 ubiquitination and TRAF6-TAK1 formation to inhibit NFATc1 activation (117). Jiahong et al. (118) also showed that catalpol upregulates the activity of phosphatase and tensin homologues by decreasing their ubiquitination, which in turn inhibits RANKL-induced NF-κB and AKT signalling pathways, leading to inhibition of osteoclast activity *in vivo*.

#### NFATc1,c-Fos, and MITF

NFATc1 is the main regulator of OC generation, which can induce the expression of OC maturation-specific genes such as Trap, CTR, integrinβ3 and CtsK (86, 119, 120). The activation of NFATc1 is realised by RANKL through calcium signal transduction. In the final stage of OC differentiation, NFATc1, together with Fos and Jun proteins, can induce the expression of OC-specific genes, such as DC-STAMP, Trap, CTR, CtsK and integrinβ3, as well as PU.1 and microphthalmia transcription factors (MITF) (121, 122).

MITF regulates the development and activity of several cell lineages, including OCs. The isoform MITF-E, which is significantly upregulated in developing OCs, is induced by RANKL and is critically important for OC genesis (123). Moreover, MITF-E has more recently been shown to regulate OC genesis by modulating the activity of NFATc1 (124). MITF is activated downstream of p38 MAP kinase, which is activated due to RANKL signalling. MITF is crucial to the expression of genes encoding the OC-specific proteins Trap and CtsK.

#### CREB

Calcium (Ca2+) signalling is essential for various cellular responses and higher biological functions. Ca2+/calmodulin dependent kinases (CaMKs) and the phosphatase calcineurin activate distinct downstream pathways that are mediated by the transcription factors cAMP response element (CRE)-binding protein (CREB) and nuclear factor of activated T cells (NFAT), respectively (125).

CREB and NFATc1 co-induced the expression of specific genes in differentiated OCs. Through the biphasic regulation of osteoclastic bone resorption, the CaMK-CREB pathway enhances the induction of NFATc1 on the one hand, and the expression of NFATc1 is induced on the other hand, which promotes the regulation of NFATc1-dependent genes (126). When extracellular RANKL binds to RANK on the surface of OCs, it triggers an increase in intracellular Ca2+ concentration (^[^Ca2+] I), which stimulates a series of Ca2+/calmodulin-binding proteins (CaMKs and Cacineurin), such as CaMKs and Calcineurin (CN). CaMKIV is mainly involved in the phosphorylation of CREB and induces the expression of c-Fos. C-Fos plays an indispensable role in inducing NFATc1 (120). NFATc1 is activated by calcineurin-mediated dephosphorylation and translocated to the nucleus to induce osteoclast-specific gene expression. Recent evidence suggests that CREB is phosphorylated through the MAPK signal pathway (127).

Puerariae radix, the dried root of Pueraria lobate Ohwi, can significantly inhibit the activation of cAMP response element binding protein (CREB) and the induction of peroxisome proliferator-activated receptor γ coactivator 1 β (PGC1 β), thus inhibiting the differentiation and formation of OCs, decrease the bone resorption activity of OCs and down-regulate the expression of OC differentiation marker genes (128). BCAP promotes OC differentiation by regulating the p38-dependent CREB signalling pathway (21). Wang Lufei et al. (129) found for the first time that Dopamine can trigger the D2-like receptors D2R/cAMP/PKA/CREB pathway to inhibit OC differentiation. CAMP/PKA is thought to negatively regulate OC generation through phosphorylation and inactivation of NFATC1 and crosstalk with Wnt or Ca2+/CaMK signals (130, 131).

#### Pu.1

Pu.1 knockout mice have defects in both macrophage and OC differentiation, making PU.1 one of the earliest markers of OC lineage (132). PU.1 is a transcription factor that affects OC generation by cooperating with IRF8 or NFATc1 (121) to establish specific types of enhancers in OCPs and mature OCs. Carey HA et al. (122) determined that EOMES is a cofactor that regulates the expression of the NFATc1 site and affects the differentiation of OCs by interacting with PU.1 and MITF.

#### Nicotinamide phosphoribosyltransferase

NAMPT is a key rate-limiting enzyme in the nicotinamide adenine dinucleotide (NAD) biosynthesis pathway (133) and plays an important role in NAD synthesis, apoptosis and inflammation (134). The disorder of NAMPT is associated with RA. In the mouse model of CIA, Busso et al. (135) found that the NAMPT inhibitor FK866 can effectively reduce the severity of arthritis and disease progression. In Ly6C^high^ monocytes, selective knockout of siRNA of NAMPT also inhibited the progress of CIA (136). LI et al. (119) systematically confirmed that Nampt is a genetic risk factor and potential therapeutic target for RA in CIA mice and cell models. In the CIA mouse model, Nampt deficient mice showed inflammatory bone destruction and a decreased OC generation ability of primary BMMs and RAW264.7 cells with NAMPT deficiency. This study proved that NAMPT is an important epigenetic regulator of OC generation in RA.

#### silence information regulator 1

Sirtuin1 (SIRT1), a member of the Sirtuin protein family, is an enzyme responsible for the deacetylation of proteins regulated by cells. SIRT1 is a NAD-dependent enzyme that uses NAD substrates to remove acetyl groups from proteins. It is in the nucleus and can also act on the cytoplasmic target (137). Sirt1 can deacetylate the lysine at position 26 of histone H1, 9 of H3 and 16 of H4, and mainly regulates NF-κB, HIFl α, and Notch in the cytoplasm.

Sirt1 regulates the expression of NF-κB by maintaining the state of OCP deacetylation (138), thus regulating the generation of OCs. In the CIA rat model, Sirt1 reduces the transcriptional activity of NF-κB through deacetylation of p65 and p300 (139), inhibits the synthesis and secretion of inflammatory factors mediated by inhibiting activation of NF-κB signal pathway, and alleviates symptoms of arthritis. Edwards et al. (140) found that in OC-specific Sirt1 knockout mice, the bone volume decreased, the acetylation level of primary OCs increased, and OCPs formed more TRAP+ cells than the primary bone marrow cells of control mice. In the absence of Sirt1, excessive NF-κB signal activation leads to increased OC generation, which decreases bone mass. The inhibition of the NF-κB signal pathway can reverse the decrease of bone mass, indicating that Sirt1 can maintain normal bone formation by inhibiting the NF-κB signal pathway. These studies proved that NAMPT is an important epigenetic regulator of OC generation in RA.

Hah et al. (141) showed that Sirt1-deficient macrophages increased the polarisation of M1 through hyperacetylation, caused over-activation of the NF-κB pathway, and increased the production of pro-inflammatory cytokine. The deletion of Sirt1 leads to the formation of multinucleated TRAP+ cells (OC), which aggravates the occurrence and development of RA. The macrophages of Sirt1 transgenic mice showed increased polarisation of M2 macrophages and decreased polarisation of M1 phenotypic macrophages, accompanied by an increase in anti-inflammatory factors and a decrease in pro-inflammatory factors. Therefore, Sirt1 inhibits RA inflammation and indirectly reduces OC generation by regulating the proportion of M1/M2 polarisation of macrophages to promote bone remodelling. This study shows that SIRT1 plays a protective role in RA.

#### Küppel-like factors2

Küppel-like factors (KLF) are a family of DNA-binding zinc finger proteins that can be used as transcriptional activators or repressors. Previous studies have found that KLF2 is key in promoting vascular integrity, pulmonary function, cardiovascular development and atherosclerotic protective properties (142–144).

In terms of OCs, KLF2 weakens OC generation through several complementary mechanisms. On the one hand, KLF2 inhibits the activity of NF-κB through direct interaction with epigenetic regulators p300 and PCAF, which are important coactivators of directed transcription of NF-κB (145, 146). However, in RA, the deletion of KLF2 decreased the overall expression of MMP-9 and inflammatory cytokine (147). The knockout of KLF2 significantly increased the enrichment of active histone markers H3K9Ac and H4K8Ac, and the enrichment of histone acetylase transfer (HAT enzyme) in MMP9 gene enrichment sites P300 and PCAF (148), resulting in increased OC generation and more aggressive disease progression.

In mBSA and IL-1-induced arthritis mouse models, KLF2 regulates monocyte differentiation and function, and monocytes derived from the bone marrow of KLF2 hemizygotes mice are more likely to differentiate into OCs than cells obtained from wild-type(WT) mice *in vitro (* 149). The number of TRAP-stained cells in the KLF2+/-population was significantly higher than that in the WT population, and the expression levels of OC-specific proteins such as MMP-9, NFATc1, and vATPase in the bone marrow cells of RA mice induced by K/BxN serum were also higher than those in the WT population’s cells (150). Concurrently, the expression level of pro-inflammatory molecules in serum was higher, and that of anti-inflammatory cytokine was lower. These results suggest that KLF2 may play a protective role in bone and surrounding tissue by reducing inflammation of arthritic joints.

To sum up, KLF2 plays an important role in reducing inflammation and inhibiting the differentiation and function of OCs in RA, so it may be a target for OCs in treating RA.

#### Merlot

Yamakawa (151) and other researchers’ studies have found that the newly discovered gene Merlot, which can encode highly conserved but uncharacteristic proteins in vertebrates, is an important regulator for terminating OC generation. Stimulated by RANKL, Merlot significantly induced, inhibited the differentiation of OCs, and induced their apoptosis.

Merlot knockout mice showed low bone mass due to the increase in OCs number and bone resorption; Relative to the WT cells, the formation of multinucleated OCs in Merlot knockout RAW264.7 cells was faster, the mRNA expression of OC-specific genes (Acp5, Mmp9, CtsK and Otcar) significantly increased, and the life span of OCs was prolonged.

OCP overexpressing Merlot can not differentiate into mature OCs, while deficiency of Merlot leads to excessive nucleation and prolonged survival of OCs. Along with the continuous nuclear localisation of NFATc1 and the decrease of glycogen synthase kinase-3β (GSK3β) activity, Merlot regulates the lifespan of OCs by inhibiting the differentiation of NFATc1-GSK3 axis and promoting OCs apoptosis at the same time.

They also found that Merlot knockout inhibited caspase-3-mediated apoptosis in OCs, and deletion of Merlot led to down-regulation of apoptosis-promoting cascades, thereby promoting OCs survival.

**Figure 3:**
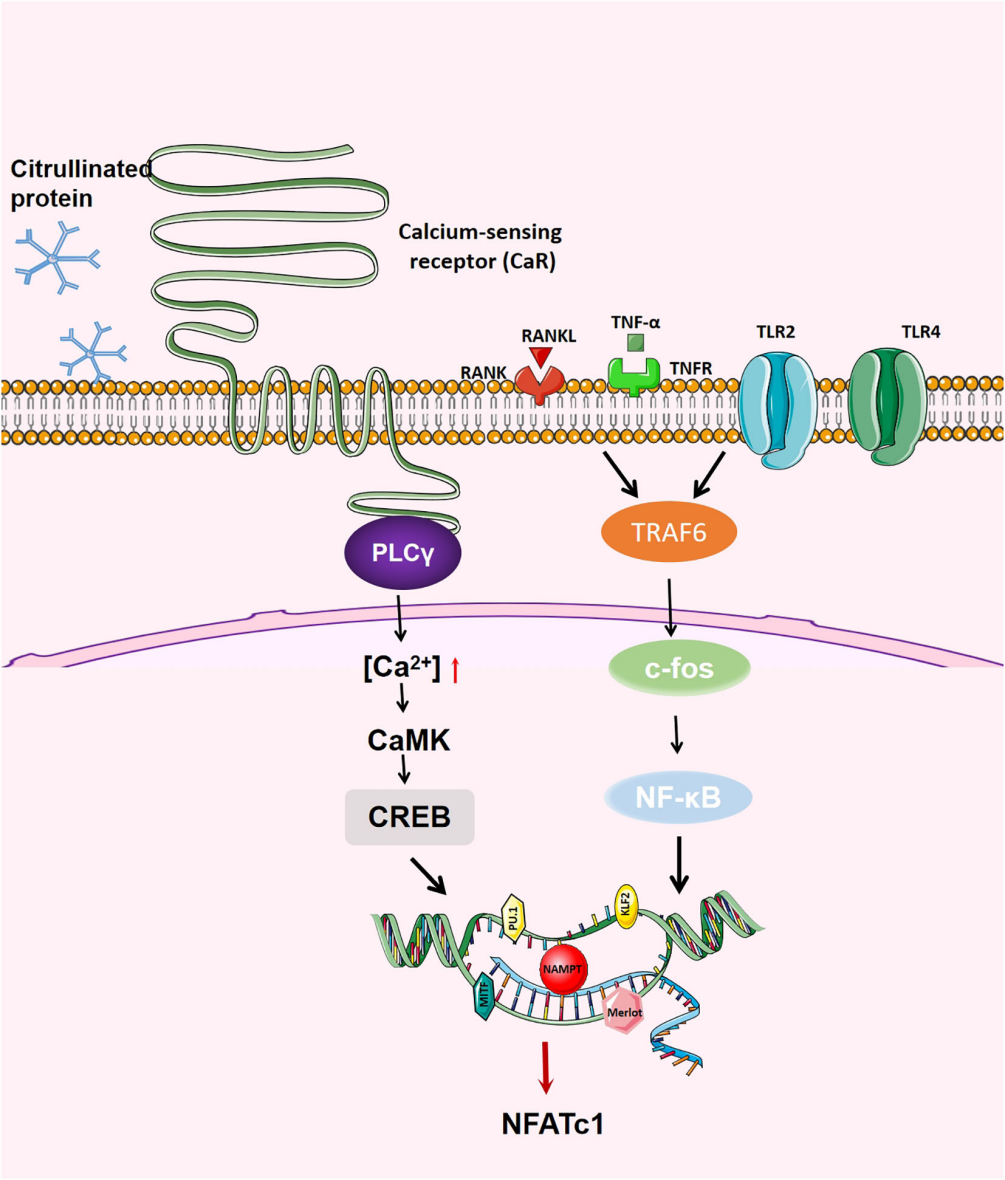
Generation regulation of OCs. When RANK binds to RANKL, on the one hand, RANK recruits TRAF6, activates NF- κB, JNK,p38,c-fos and AP-1; on the other hand, RANK increases the activity of intracellular calcium and activates calcium regulatory neuroenzymes through the Btk/Tec pathway, which promotes the production of phospholipase C (PLC) to mediate the release of intracellular calcium, regulating the activity of CREB and affecting the transcription of various transcription factors.NFATc1 is the main transcription factor in OC differentiation, inducing OC-specific target genes in the nucleus.The activation of NFATc1 is induced by the above two pathways, thus promoting the formation of OC.

These results show that Merlot can terminate the function related to OC genesis by activating the apoptosis pathway, indicating that it plays an important role in regulating OC lifespan. (**Figure 3**)

### Environmental Factors

Aromatic hydrocarbon receptor (AhR) is a ligand-activated transcription factor of the PAS family, which was initially associated with the heterophytic metabolism of environmental pollutants. However, recent studies have clarified the role of AhR in immune regulation and bone remodelling (152). Tobacco smoke is a recognised risk factor for bone destruction diseases, including RA, which contains many environmental toxins, including polycyclic aromatic hydrocarbons (PAH) such as benzopyrene (BaP) and dioxins such as TCDD. BaP and TCDD are both mature AhR agonists (153). Low concentrations of BaP may promote OC generation (154), while a high concentration of BaP may reduce the transformation of the RAW264.7 cell line to OCs. Moreover, AhR is related to RANKL-induced OC generation, in which RANKL induces the expression of AhR-related genes Cyp1a1 and Cyp1a2 (155). Therefore, increasing aromatic hydrocarbons in the environment will increase the interaction between AhR and RANKL in the active body, promote OC generation, and aggravate bone destruction in RA (156).

Oral administration of tetraampuline (which can activate AhR ubiquitin) in CIA rats reduced the phosphorylation of syk-positive cells and the number of OCs and bone erosion in the epiphyseal region of the proximal tibia by inhibiting the activation of NFATc1 (157). In addition, the AhR signal pathway can also regulate the NF-κB, Wnt, and MAP kinase pathways, affect the function and differentiation of OCs, and lead to changes in bone remodelling (158–161). Donate et al. (162) used transcriptome analysis to show that miRNA-132 was specifically induced in Th17 cells in the presence of an AhR agonist or medium-rich in tobacco smoke, and the induced miRNA-132 was encapsulated in the extracellular vesicles produced by Th17 and increased OC generation by down-regulating COX2 as a pro-inflammatory mediator. In the arthritis model of mice with a miRNA-132 knockout, the number of OCs in the joints decreased. Clinically, the level of miRNA-132 expressed by T cells in patients with RA was higher than that in healthy controls, indirectly affecting the inflammatory environment of OC generation.

Overall, these results indicate that tobacco smoke can aggravate the differentiation of OCs in RA and promote the progress of the disease.

## Treatment of targeted osteoclast in RA

In recent years, the treatment of RA has made great progress; however, most RA patients are experiencing non-optimal responses to the current treatment, especially those aimed at bone injuries and joint deformities. Most RA treatments focus on inhibiting the proliferation of abnormally activated immune cells rather than directly targeting bone destruction. Even if the inflammation is reduced, structural damage may continue to develop, as immunosuppressive drugs target neither OCs nor FLS, which expresses RANKL. The role of OCs in mediating bone erosion of RA is direct and obvious, so the targeting study of OCs is expected to produce an effective solution to bone destruction in patients with RA.

### Targeted KLF2

Currently, most drugs aim to reduce bone resorption or promote bone formation but targeting KLF2 may lead to dual-functional therapeutic effects. In order to test the therapeutic potential of KLF2, Rolph (148) used a pharmacological compound histone deacetylase inhibitor(HDACi) to induce KLF2 and found that the expression of KLF2 in myeloid cells increased *in vitro* and *in vivo*. The induction of KLF2 significantly reduced the differentiation of OCs and decreased MMP expression. However, the detailed signalling mechanism of this cytokine in RA bone destruction needs to be further studied.

### Targeted Tyro3TK

In RA, it is particularly important to inhibit the invasiveness of OCs. Tyro3TK was initially a therapeutic target for tumours (163). Increasing studies have found that it also plays a key role in autoimmune diseases (164, 165). Tyro3TK plays a unique role in regulating the differentiation of CD14+CD16-monocytes into OCs, while Tyro3TK has a more obvious inhibitory effect on CD14+CD16- monocytes, which is one of the therapeutic targets of bone destruction in RA (43).

### Targeted Sirt1

Sirt1 has been shown to inhibit OC differentiation through negative regulation of NF-κB and positive regulation of FOXO transcription factor (43). Encouragingly, a recent randomised placebo-controlled trial showed that resveratrol, a first-generation Sirt agonist, increased bone mineral density and markers of bone formation in obese men for 16 weeks (55). Drugs targeting Sirt1 also show hope for treating other types of bone diseases and can play a role in the treatment of RA.

### Targeted RANKL

The important role of RANKL in RA-related bone lesions is self-evident. Denosumab is a humanised anti-RANKL monoclonal antibody, which competitively binds to RANK and inhibits the role of RANKL, thus inhibiting OC generation (166). Denosumab can effectively increase bone mineral density and prevent osteoporosis in patients with RA (167–169). In a multicentre, randomised, double-blind, parallel-group, placebo-controlled clinical study conducted in Japan, it was found that Denosumab inhibits the progression of joint destruction, increases BMD, and is well tolerated in patients with Ra taking csDMaRD (170).

### Targeted TNF-α

TNF-α inhibition therapy is used to reduce the inflammation of RA (171). These treatments can reduce the differentiation and activation of OCs by reducing the expression of RANKL on B cells and the level of serum-soluble RANKL.

TNF-α plays an important role in the pathogenesis of RA. On the one hand, TNF-α can induce TRAP-positive cells in the absence of RANKL *via* the NF-κB pathway. On the other hand, TNF-α induces RANK expression through OCP. In addition, TNF-α and RANKL synergistically induce OC generation through TRAF-3 signalling in a TRAF-6-independent pathway. Importantly, TNF-α can indirectly regulate OCs through various stimuli of stromal cells. It was found that the number of OCP in blood and the ability of monocytes to differentiate into OCP in RA patients decreased after the use of TNF-α antagonist and effectively prevented the decrease of bone mineral density in the hip and lumbar vertebrae, which had a good effect on active RA patients with reduced bone mass (172, 173).

Iguratimod is a small molecular drug shown to have a significant osteoprotective effect on patients with RA. The inhibitory effect of Iguratimod is achieved by inhibiting RANKL and TNF-α pathway (174). Iguratimod also inhibited TNF-α-induced OC generation *in vitro* and reduced the expression of genes related to OC generation by disrupting the late nuclear transfer of NF-κB, thus reducing OC generation in RA patients (175). These results suggest that Iguratimod can be a unique choice for treating RA, especially in preventing bone loss.

## Conclusion

OCs are the most direct factor mediating bone destruction in RA. An in-depth understanding of their activation mechanism and predictive indexes can provide an effective basis for preventing joint deformities, early diagnosis of bone destruction and improving the quality of life of patients with RA. The excessive activation of OCs is the result of multiple factors. Under the stimulation of epigenetic disorders and transcriptional regulators, DNA methylation, histone acetylation, and other processes promote the expression of RANKL and the production of OCs.In recent years, discoveries about OCs functionality have been growing, such as the discovery of the gene Merlot, which promotes OC apoptosis. The up-regulation of Tyro3TK promotes OC generation, and KLF2 inhibits OC differentiation in RA, which provides new evidence for understanding the mechanism of RA-related bone destruction. However, these mechanisms and trials require further validation. Understanding the progress of these regulatory mechanisms will help us better understand the pathogenesis of RA and develop new diagnostic, therapeutic and prognostic strategies.

Given the clinical characteristics of RA, we have summarised the targeted therapy for OCs through different internal pathways, but the internal mechanism of many aspects remains elusive. We believe that combining targeted OCs and anti-inflammatory agents are promising for better clinical outcomes.

## Data availability statement

The original contributions presented in the study are included in the article/supplementary material. Further inquiries can be directed to the corresponding author.

## Author contributions

QN and JG wrote the manuscript. LW collected the references. JL drew the figures. LZ reviewed and revised the manuscript. All authors contributed to the article and approved the submitted version.

## Funding

Financial support for this research was provided by the National Natural Science Foundation of China (81202356, 81771768 and 81771292), Shanxi Provincial Key Research and Development Project (201803D31136), and the applied basic research project of Shanxi Science and Technology Department (201901D111416).

## Acknowledgments

We would like to thank Editage (www.editage.cn) for English language editing.

## Conflict of interest

The authors declare that the research was conducted in the absence of any commercial or financial relationships that could be construed as a potential conflict of interest.

## Publisher’s note

All claims expressed in this article are solely those of the authors and do not necessarily represent those of their affiliated organizations, or those of the publisher, the editors and the reviewers. Any product that may be evaluated in this article, or claim that may be made by its manufacturer, is not guaranteed or endorsed by the publisher.

## References

[1] Feng X McDonald JM . Disorders of bone remodeling. Annu Rev Pathol (2011) 6:121–45. doi: 10.1146/annurev-pathol-011110-130203 PMC3571087 20936937

[2] Li H Hong S Qian J Zheng Y Yang J Yi Q . Cross talk between the bone and immune systems: Osteoclasts function as antigen-presenting cells and activate CD4+ and CD8+ T cells. Blood (2010) 116(2):210–7. doi: 10.1182/blood-2009-11-255026 PMC2910608 20304810

[3] Sugatani T Hruska KA . Impaired micro-RNA pathways diminish osteoclast differentiation and function. J Biol Chem (2009) 284(7):4667–78. doi: 10.1074/jbc.M805777200 PMC2640963 19059913

[4] Komano Y Nanki T Hayashida K Taniguchi K Miyasaka N . Identification of a human peripheral blood monocyte subset that differentiates into osteoclasts. Arthritis Res Ther (2006) 8(5):R152. doi: 10.1186/ar2046 16987426 PMC1779441

[5] Soysa NS Alles N Aoki K Ohya K . Osteoclast formation and differentiation: An overview. J Med Dent Sci (2012) 59(3):65–74. 23897045

[6] Kurotaki D Yoshida H Tamura T . Epigenetic and transcriptional regulation of osteoclast differentiation. Bone (2020) 138:115471. doi: 10.1016/j.bone.2020.115471 32526404

[7] Lavin Y Winter D Blecher-Gonen R David E Keren-Shaul H Merad M . Tissue-resident macrophage enhancer landscapes are shaped by the local microenvironment. Cell (2014) 159(6):1312–26. doi: 10.1016/j.cell.2014.11.018 PMC4437213 25480296

[8] Gosselin D Link VM Romanoski CE Fonseca GJ Eichenfield DZ Spann NJ . Environment drives selection and function of enhancers controlling tissue-specific macrophage identities. Cell (2014) 159(6):1327–40. doi: 10.1016/j.cell.2014.11.023 PMC4364385 25480297

[9] Jung Y Wang J Song J Shiozawa Y Wang J Havens A . Annexin II expressed by osteoblasts and endothelial cells regulates stem cell adhesion, homing, and engraftment following transplantation. Blood (2007) 110(1):82–90. doi: 10.1182/blood-2006-05-021352 17360942 PMC1896130

[10] Coxon FP Taylor A . Vesicular trafficking in osteoclasts. Semin Cell Dev Biol (2008) 19(5):424–33. doi: 10.1016/j.semcdb.2008.08.004 18768162

[11] Mulari M Vaaraniemi J Vaananen HK . Intracellular membrane trafficking in bone resorbing osteoclasts. Microsc Res Tech (2003) 61(6):496–503. doi: 10.1002/jemt.10371 12879417

[12] Xu J Cheng T Feng HT Pavlos NJ Zheng MH . Structure and function of V-ATPases in osteoclasts: Potential therapeutic targets for the treatment of osteolysis. Histol histopathol (2007) 22(4):443. doi: 10.14670/HH-22.443 17290355

[13] Schett G Gravallese E . Bone erosion in rheumatoid arthritis: Mechanisms, diagnosis and treatment. Nat Rev Rheumatol (2012) 8(11):656–64. doi: 10.1038/nrrheum.2012.153 PMC4096779 23007741

[14] Ono T Nakashima T . Recent advances in osteoclast biology. Histochem Cell Biol (2018) 149(4):325–41. doi: 10.1007/s00418-018-1636-2 29392395

[15] Adamopoulos IE Mellins ED . Alternative pathways of osteoclastogenesis in inflammatory arthritis. Nat Rev Rheumatol (2015) 11(3):189–94. doi: 10.1038/nrrheum.2014.198 PMC4346500 25422000

[16] Gravallese EM Harada Y Wang JT Gorn AH Thornhill TS Goldring SR . Identification of cell types responsible for bone resorption in rheumatoid arthritis and juvenile rheumatoid arthritis. Am J Pathol (1998) 152(4):943–51. doi: 10.1038/nrrheum.2012.171 PMC1858257 9546355

[17] Brzustewicz E Bryl E . The role of cytokines in the pathogenesis of rheumatoid arthritis–practical and potential application of cytokines as biomarkers and targets of personalized therapy. Cytokine (2015) 76(2):527–36. doi: 10.1016/j.cyto.2015.08.260 26321413

[18] Ono T Takayanagi H . Osteoimmunology in bone fracture healing. Curr Osteoporos Rep (2017) 15(4):367–75. doi: 10.1007/s11914-017-0381-0 28647888

[19] Cong Q Jia H Li P Qiu S Yeh J Wang Y . p38alpha MAPK regulates proliferation and differentiation of osteoclast progenitors and bone remodeling in an aging-dependent manner. Sci Rep (2017) 7:45964. doi: 10.1038/srep45964 28382965 PMC5382695

[20] Kim JH Kim N . Regulation of NFATc1 in osteoclast differentiation. J Bone Metab (2014) 21(4):233–41. doi: 10.11005/jbm.2014.21.4.233 PMC4255043 25489571

[21] Kim JH Kim K Kim I Seong S Lee KB Kim N . BCAP promotes osteoclast differentiation through regulation of the p38-dependent CREB signaling pathway. Bone (2018) 107:188–95. doi: 10.1016/j.bone.2017.12.005 29223746

[22] Ross F P. M-CSF . C-fms, and signaling in osteoclasts and their precursors. Ann N Y Acad Sci (2006) 1068:110–6. doi: 10.1196/annals.1346.014 16831911

[23] Lee MS Kim HS Yeon JT Choi SW Chun CH Kwak HB . GM-CSF regulates fusion of mononuclear osteoclasts into bone-resorbing osteoclasts by activating the Ras/ERK pathway. J Immunol (2009) 183(5):3390–9. doi: 10.4049/jimmunol.0804314 19641137

[24] Mukherjee PM Wang CJ Chen IP Jafarov T Olsen BR Ueki Y . Cherubism gene Sh3bp2 is important for optimal bone formation, osteoblast differentiation, and function. Am J Orthod Dentofacial Orthop (2010) 138(2):140–141, 140-141. doi: 10.1016/j.ajodo.2010.04.004 PMC3268358 20691350

[25] Rivollier A Mazzorana M Tebib J Piperno M Aitsiselmi T Rabourdin-Combe C . Immature dendritic cell transdifferentiation into osteoclasts: A novel pathway sustained by the rheumatoid arthritis microenvironment. Blood (2004) 104(13):4029–37. doi: 10.1182/blood-2004-01-0041 15308576

[26] Nomura K Kuroda S Yoshikawa H Tomita T . Inflammatory osteoclastogenesis can be induced by GM-CSF and activated under TNF immunity. Biochem Biophys Res Commun (2008) 367(4):881–7. doi: 10.1016/j.bbrc.2008.01.023 18201554

[27] Ospelt C Brentano F Rengel Y Stanczyk J Kolling C Tak PP . Overexpression of toll-like receptors 3 and 4 in synovial tissue from patients with early rheumatoid arthritis: Toll-like receptor expression in early and longstanding arthritis. Arthritis Rheum (2008) 58(12):3684–92. doi: 10.1002/art.24140 19035519

[28] Seibl R Birchler T Loeliger S Hossle JP Gay RE Saurenmann T . Expression and regulation of toll-like receptor 2 in rheumatoid arthritis synovium. Am J Pathol (2003) 162(4):1221–7. doi: 10.1016/S0002-9440(10)63918-1 PMC1851232 12651614

[29] Takami M Kim N Rho J Choi Y . Stimulation by toll-like receptors inhibits osteoclast differentiation. J Immunol (2002) 169(3):1516–23. doi: 10.4049/jimmunol.169.3.1516 12133979

[30] Lu Q Jiang C Hou J Qian H Chu F Zhang W . Patchouli alcohol modulates the pregnancy X Receptor/Toll-like receptor 4/Nuclear factor kappa b axis to suppress osteoclastogenesis. Front Pharmacol (2021) 12:684976. doi: 10.3389/fphar.2021.684976 34177594 PMC8227438

[31] Joosten LA Abdollahi-Roodsaz S Heuvelmans-Jacobs M Helsen MM van den Bersselaar LA Oppers-Walgreen B . T Cell dependence of chronic destructive murine arthritis induced by repeated local activation of toll-like receptor-driven pathways: Crucial role of both interleukin-1beta and interleukin-17. Arthritis Rheum (2008) 58(1):98–108. doi: 10.1002/art.23152 18163514

[32] Abdollahi-Roodsaz S Joosten LA Helsen MM Walgreen B van Lent PL van den Bersselaar LA . Shift from toll-like receptor 2 (TLR-2) toward TLR-4 dependency in the erosive stage of chronic streptococcal cell wall arthritis coincident with TLR-4-mediated interleukin-17 production. Arthritis Rheum (2008) 58(12):3753–64. doi: 10.1002/art.24127 19035506

[33] Kim SJ Chen Z Chamberlain ND Essani AB Volin MV Amin MA . Ligation of TLR5 promotes myeloid cell infiltration and differentiation into mature osteoclasts in rheumatoid arthritis and experimental arthritis. J Immunol (2014) 193(8):3902–13. doi: 10.4049/jimmunol.1302998 PMC4185216 25200955

[34] Alzabin S Kong P Medghalchi M Palfreeman A Williams R Sacre S . Investigation of the role of endosomal toll-like receptors in murine collagen-induced arthritis reveals a potential role for TLR7 in disease maintenance. Arthritis Res Ther (2012) 14(3):R142. doi: 10.1186/ar3875 22691272 PMC3446525

[35] Duffau P Menn-Josephy H Cuda CM Dominguez S Aprahamian TR Watkins AA . Promotion of inflammatory arthritis by interferon regulatory factor 5 in a mouse model. Arthritis Rheumatol (2015) 67(12):3146–57. doi: 10.1002/art.39321 PMC4661118 26315890

[36] Kim KW Kim BM Won JY Lee KA Kim HR Lee SH . Toll-like receptor 7 regulates osteoclastogenesis in rheumatoid arthritis. J Biochem (2019) 166(3):259–70. doi: 10.1093/jb/mvz033 31086948

[37] Lemke G . Phosphatidylserine is the signal for TAM receptors and their ligands. Trends Biochem Sci (2017) 42(9):738–48. doi: 10.1016/j.tibs.2017.06.004 PMC5600686 28734578

[38] Rothlin CV Carrera-Silva EA Bosurgi L Ghosh S . TAM receptor signaling in immune homeostasis. Annu Rev Immunol (2015) 33:355–91. doi: 10.1146/annurev-immunol-032414-112103 PMC4491918 25594431

[39] Nakamura YS Hakeda Y Takakura N Kameda T Hamaguchi I Miyamoto T . Tyro 3 receptor tyrosine kinase and its ligand, Gas6, stimulate the function of osteoclasts. Stem Cells (1998) 16(3):229–38. doi: 10.1002/stem.160229 9617898

[40] Katagiri M Hakeda Y Chikazu D Ogasawara T Takato T Kumegawa M . Mechanism of stimulation of osteoclastic bone resorption through Gas6/Tyro 3, a receptor tyrosine kinase signaling, in mouse osteoclasts. J Biol Chem (2001) 276(10):7376–82. doi: 10.1074/jbc.M007393200 11084030

[41] Kawaguchi H Katagiri M Chikazu D . Osteoclastic bone resorption through receptor tyrosine kinase and extracellular signal-regulated kinase signaling in mature osteoclasts. Mod Rheumatol (2004) 14(1):1–5. doi: 10.3109/s10165-003-0257-2 17028797

[42] Ruiz-Heiland G Zhao Y Derer A Braun T Engelke K Neumann E . Deletion of the receptor tyrosine kinase Tyro3 inhibits synovial hyperplasia and bone damage in arthritis. Ann Rheum Dis (2014) 73(4):771–9. doi: 10.1136/annrheumdis-2012-202907 23632195

[43] Xue J Xu L Zhu H Bai M Li X Zhao Z . CD14(+)CD16(-) monocytes are the main precursors of osteoclasts in rheumatoid arthritis *via* expressing Tyro3TK. Arthritis Res Ther (2020) 22(1):221. doi: 10.1186/s13075-020-02308-7 32958023 PMC7507256

[44] Peeters M Rahbech A Thor SP . TAM-ing T cells in the tumor microenvironment: Implications for TAM receptor targeting. Cancer Immunol Immunother (2020) 69(2):237–44. doi: 10.1007/s00262-019-02421-w PMC7000491 31664482

[45] O’Donnell K Harkes IC Dougherty L Wicks IP . Expression of receptor tyrosine kinase axl and its ligand Gas6 in rheumatoid arthritis: Evidence for a novel endothelial cell survival pathway. Am J Pathol (1999) 154(4):1171–80. doi: 10.1016/S0002-9440(10)65369-2 PMC1866575 10233855

[46] Danks L Komatsu N Guerrini MM Sawa S Armaka M Kollias G . RANKL expressed on synovial fibroblasts is primarily responsible for bone erosions during joint inflammation. Ann Rheum Dis (2016) 75(6):1187–95. doi: 10.1136/annrheumdis-2014-207137 26025971

[47] Zizzo G Hilliard BA Monestier M Cohen PL . Efficient clearance of early apoptotic cells by human macrophages requires M2c polarization and MerTK induction. J Immunol (2012) 189(7):3508–20. doi: 10.4049/jimmunol.1200662 PMC3465703 22942426

[48] Degboe Y Rauwel B Baron M Boyer JF Ruyssen-Witrand A Constantin A . Polarization of rheumatoid macrophages by TNF targeting through an IL-10/STAT3 mechanism. Front Immunol (2019) 10:3. doi: 10.3389/fimmu.2019.00003 30713533 PMC6345709

[49] Zhao GJ Zheng JY Bian JL Chen LW Dong N Yu Y . Growth arrest-specific 6 enhances the suppressive function of CD4(+)CD25(+) regulatory T cells mainly through axl receptor. Mediators Inflammation (2017) 2017:6848430. doi: 10.1155/2017/6848430 PMC5320320 28270700

[50] Sargin G Kose R Senturk T . Relationship between bone mineral density and anti-citrullinated protein antibody and rheumatoid factor in patients with rheumatoid arthritis. Eur J Rheumatol (2019) 6(1):29–33. doi: 10.5152/eurjrheum.2018.18099 30973322 PMC6459337

[51] Orsolini G Caimmi C Viapiana O Idolazzi L Fracassi E Gatti D . Titer-dependent effect of anti-citrullinated protein antibodies on systemic bone mass in rheumatoid arthritis patients. Calcif Tissue Int (2017) 101(1):17–23. doi: 10.1007/s00223-017-0253-8 28246933

[52] Hafstrom I Ajeganova S Forslind K Svensson B . Anti-citrullinated protein antibodies are associated with osteopenia but not with pain at diagnosis of rheumatoid arthritis: Data from the BARFOT cohort. Arthritis Res Ther (2019) 21(1):45. doi: 10.1186/s13075-019-1833-y 30717793 PMC6360733

[53] Harre U Keppeler H Ipseiz N Derer A Poller K Aigner M . Moonlighting osteoclasts as undertakers of apoptotic cells. Autoimmunity (2012) 45(8):612–9. doi: 10.3109/08916934.2012.719950 22978425

[54] Harre U Lang SC Pfeifle R Rombouts Y Frühbeißer S Amara K . Glycosylation of immunoglobulin G determines osteoclast differentiation and bone loss. Nat Commun (2015) 6:6651. doi: 10.1038/ncomms7651 25825024 PMC4389255

[55] Seeling M Hillenhoff U David JP Schett G Tuckermann J Lux A . Inflammatory monocytes and fcgamma receptor IV on osteoclasts are critical for bone destruction during inflammatory arthritis in mice. Proc Natl Acad Sci USA (2013) 110(26):10729–34. doi: 10.1073/pnas.1301001110 PMC3696830 23754379

[56] Liu X Chen Z Lan T Liang P Tao Q . Upregulation of interleukin-8 and activin a induces osteoclastogenesis in ameloblastoma. Int J Mol Med (2019) 43(6):2329–40. doi: 10.3892/ijmm.2019.4171 PMC6488175 31017256

[57] Krishnamurthy A Joshua V Haj HA Jin T Sun M Vivar N . Identification of a novel chemokine-dependent molecular mechanism underlying rheumatoid arthritis-associated autoantibody-mediated bone loss. Ann Rheum Dis (2016) 75(4):721–9. doi: 10.1136/annrheumdis-2015-208093 PMC4819614 26612338

[58] Negishi-Koga T Gober HJ Sumiya E Komatsu N Okamoto K Sawa S . Immune complexes regulate bone metabolism through FcRgamma signalling. Nat Commun (2015) 6:6637. doi: 10.1038/ncomms7637 25824719

[59] Komatsu N Win S Yan M Huynh NC Sawa S Tsukasaki M . Plasma cells promote osteoclastogenesis and periarticular bone loss in autoimmune arthritis. J Clin Invest (2021) 131(6). doi: 10.1172/JCI143060 PMC7954598 33720039

[60] Meednu N Zhang H Owen T Sun W Wang V Cistrone C . Production of RANKL by memory b cells: A link between b cells and bone erosion in rheumatoid arthritis. Arthritis Rheumatol (2016) 68(4):805–16. doi: 10.1002/art.39489 PMC4956406 26554541

[61] Sato K Takayanagi H . Osteoclasts, rheumatoid arthritis, and osteoimmunology. Curr Opin Rheumatol (2006) 18(4):419–26. doi: 10.1097/01.bor.0000231912.24740.a5 16763464

[62] Yudoh K Matsuno H Nakazawa F Yonezawa T Kimura T . Reduced expression of the regulatory CD4+ T cell subset is related to Th1/Th2 balance and disease severity in rheumatoid arthritis. Arthritis Rheum (2000) 43(3):617–27. doi: 10.1002/1529-0131(200003)43:3<617::AID-ANR19>3.0.CO;2-B 10728756

[63] Gigliotti CL Boggio E Clemente N . ICOS-ligand triggering impairs osteoclast differentiation and function *In vitro* and *in vivo* . J Immunol (2016) 197(10):3905–16. doi: 10.4049/jimmunol.1600424 27798154

[64] Sato K Suematsu A Okamoto K Shivakumar Y Toth E Sblattero D . Th17 functions as an osteoclastogenic helper T cell subset that links T cell activation and bone destruction. J Exp Med (2006) 203(12):2673–82. doi: 10.1084/jem.20061775 PMC2118166 17088434

[65] Horwood NJ Elliott J Martin TJ Gillespie MT . IL-12 alone and in synergy with IL-18 inhibits osteoclast formation *in vitro* . J Immunol (2001) 166(8):4915–21. doi: 10.4049/jimmunol.166.8.4915 11290769

[66] Hong MH Williams H Jin CH Pike JW . The inhibitory effect of interleukin-10 on mouse osteoclast formation involves novel tyrosine-phosphorylated proteins. J Bone Miner Res (2000) 15(5):911–8. doi: 10.1359/jbmr.2000.15.5.911 10804021

[67] Dong C . Diversification of T-helper-cell lineages: finding the family root of IL-17-producing cells. Nat Rev Immunol (2006) 6(4):329–33. doi: 10.1038/nri1807 16557264

[68] Weaver CT Harrington LE Mangan PR Gavrieli M Murphy KM . Th17: An effector CD4 T cell lineage with regulatory T cell ties. Immunity (2006) 24(6):677–88. doi: 10.1016/j.immuni.2006.06.002 16782025

[69] Nanke Y Kobashigawa T Yago T Kawamoto M Yamanaka H Kotake Sc . RANK expression and osteoclastogenesis in human monocytes in peripheral blood from rheumatoid arthritis patients. BioMed Res Int (2016) 2016:4874195. doi: 10.1155/2016/4874195 27822475 PMC5086380

[70] Komatsu N Okamoto K Sawa S Nakashima T Oh-hora M Kodama T . Pathogenic conversion of Foxp3+ T cells into TH17 cells in autoimmune arthritis. Nat Med (2014) 20(1):62–8. doi: 10.1038/nm.3432 24362934

[71] Bettelli E Carrier Y Gao W Korn T Strom TB Oukka M . Reciprocal developmental pathways for the generation of pathogenic effector TH17 and regulatory T cells. Nature (2006) 441(7090):235–8. doi: 10.1038/nature04753 16648838

[72] Andersen M Boesen M Ellegaard K Christensen R Söderström K Søe N . Synovial explant inflammatory mediator production corresponds to rheumatoid arthritis imaging hallmarks: A cross-sectional study. Arthritis Res Ther (2014) 16(3):R107. doi: 10.1186/ar4557 24886513 PMC4078218

[73] Joosten LA van De Loo FA Lubberts E Helsen MM Netea MG van Der Meer JW . An IFN-gamma-independent proinflammatory role of IL-18 in murine streptococcal cell wall arthritis. J Immunol (2000) 165(11):6553–8. doi: 10.4049/jimmunol.165.11.6553 11086098

[74] Self-Fordham JB Naqvi AR Uttamani JR Kulkarni V Nares S . MicroRNA: Dynamic regulators of macrophage polarization and plasticity. Front Immunol (2017) 8:1062. doi: 10.3389/fimmu.2017.01062 28912781 PMC5583156

[75] Soler PB Estrada-Capetillo L Izquierdo E Criado G Nieto C Municio C . Macrophages from the synovium of active rheumatoid arthritis exhibit an activin a-dependent pro-inflammatory profile. J Pathol (2015) 235(3):515–26. doi: 10.1002/path.4466 25319955

[76] Ji JD Park-Min KH Shen Z Fajardo RJ Goldring SR McHugh KP . Inhibition of RANK expression and osteoclastogenesis by TLRs and IFN-gamma in human osteoclast precursors. J Immunol (2009) 183(11):7223–33. doi: 10.4049/jimmunol.0900072 PMC2783334 19890054

[77] Cuda CM Pope RM Perlman H . The inflammatory role of phagocyte apoptotic pathways in rheumatic diseases. Nat Rev Rheumatol (2016) 12(9):543–58. doi: 10.1038/nrrheum.2016.132 PMC5297631 27549026

[78] Lin NY Beyer C Giessl A Kireva T Scholtysek C Uderhardt S . Autophagy regulates TNFalpha-mediated joint destruction in experimental arthritis. Ann Rheum Dis (2013) 72(5):761–8. doi: 10.1136/annrheumdis-2012-201671 22975756

[79] Onuora S . Bone research: Autophagy is central to joint destruction in arthritis. Nat Rev Rheumatol (2012) 8(11):633. doi: 10.1038/nrrheum.2012.171 23027031

[80] Bartok B Firestein GS . Fibroblast-like synoviocytes: Key effector cells in rheumatoid arthritis. Immunol Rev (2010) 233(1):233–55. doi: 10.1111/j.0105-2896.2009.00859.x PMC2913689 20193003

[81] Zhang X Yuan Y Pan Z Ma Y Wu M Yang J . Elevated circulating IL-17 level is associated with inflammatory arthritis and disease activity: A meta-analysis. Clin Chim Acta (2019) 496:76–83. doi: 10.1016/j.cca.2019.06.026 31271739

[82] Okamoto K Nakashima T Shinohara M Negishi-Koga T Komatsu N Terashima A . Osteoimmunology: The conceptual framework unifying the immune and skeletal systems. Physiol Rev (2017) 97(4):1295–349. doi: 10.1152/physrev.00036.2016 28814613

[83] Nishikawa K Iwamoto Y Kobayashi Y Katsuoka F Kawaguchi S Tsujita T . DNA Methyltransferase 3a regulates osteoclast differentiation by coupling to an s-adenosylmethionine-producing metabolic pathway. Nat Med (2015) 21(3):281–7. doi: 10.1038/nm.3774 25706873

[84] Yasui T Hirose J Tsutsumi S Nakamura K Aburatani H Tanaka S . Epigenetic regulation of osteoclast differentiation: Possible involvement of Jmjd3 in the histone demethylation of Nfatc1. J Bone Miner Res (2011) 26(11):2665–71. doi: 10.1002/jbmr.464 21735477

[85] Delgado-Calle J Sanudo C Fernandez AF García-Renedo R Fraga MF Riancho JA . Role of DNA methylation in the regulation of the RANKL-OPG system in human bone. Epigenetics (2012) 7(1):83–91. doi: 10.4161/epi.7.1.18753 22207352 PMC3337833

[86] Qin J Wen B Liang Y Yu W Li H . Histone modifications and their role in colorectal cancer (Review). Pathol Oncol Res (2020) 26(4):2023–33. doi: 10.1007/s12253-019-00663-8 PMC7471167 31055775

[87] Kim JH Kim K Youn BU Jin HM Kim JY Moon JB . RANKL induces NFATc1 acetylation and stability *via* histone acetyltransferases during osteoclast differentiation. Biochem J (2011) 436(2):253–62. doi: 10.1042/BJ20110062 21413932

[88] Landgraf P Rusu M Sheridan R Sewer A Iovino N Aravin A . A mammalian microRNA expression atlas based on small RNA library sequencing. Cell (2007) 129(7):1401–14. doi: 10.1016/j.cell.2007.04.040 PMC2681231 17604727

[89] Kagiya T Nakamura S . Expression profiling of microRNAs in RAW264.7 cells treated with a combination of tumor necrosis factor alpha and RANKL during osteoclast differentiation. J Periodontal Res (2013) 48(3):373–85. doi: 10.1111/jre.12017 23078176

[90] Qu B Xia X Yan M Gong K Deng S Huang G . miR-218 is involved in the negative regulation of osteoclastogenesis and bone resorption by partial suppression of p38MAPK-c-Fos-NFATc1 signaling: Potential role for osteopenic diseases. Exp Cell Res (2015) 338(1):89–96. doi: 10.1016/j.yexcr.2015.07.023 26216483

[91] Chen C Cheng P Xie H Zhou HD Wu XP Liao EY . MiR-503 regulates osteoclastogenesis *via* targeting RANK. J Bone Miner Res (2014) 29(2):338–47. doi: 10.1002/jbmr.2032 23821519

[92] Guo LJ Liao L Yang L Li Y Jiang TJ . MiR-125a TNF receptor-associated factor 6 to inhibit osteoclastogenesis. Exp Cell Res (2014) 321(2):142–52. doi: 10.1016/j.yexcr.2013.12.001 24360988

[93] Zhao C Sun W Zhang P Ling S Li Y Zhao D . miR-214 promotes osteoclastogenesis by targeting Pten/PI3k/Akt pathway. RNA Biol (2015) 12(3):343–53. doi: 10.1080/15476286.2015.1017205 PMC4615895 25826666

[94] Mizoguchi F Murakami Y Saito T Miyasaka N Kohsaka H . miR-31 controls osteoclast formation and bone resorption by targeting RhoA. Arthritis Res Ther (2013) 15(5):R102. doi: 10.1186/ar4282 24004633 PMC3978447

[95] Chen CZ Li L Lodish HF Bartel DP . MicroRNAs modulate hematopoietic lineage differentiation. Science (2004) 303(5654):83–6. doi: 10.1126/science.1091903 14657504

[96] Li T Morgan MJ Choksi S Zhang Y Kim YS Liu ZG . MicroRNAs modulate the noncanonical transcription factor NF-kappaB pathway by regulating expression of the kinase IKKalpha during macrophage differentiation. Nat Immunol (2010) 11(9):799–805. doi: 10.1038/ni.1918 20711193 PMC2926307

[97] Li YT Chen SY Wang CR Liu MF Lin CC Jou IM . Brief report: amelioration of collagen-induced arthritis in mice by lentivirus-mediated silencing of microRNA-223. Arthritis Rheum (2012) 64(10):3240–5. doi: 10.1002/art.34550 22674011

[98] Ohtsubo K Marth JD . Glycosylation in cellular mechanisms of health and disease. Cell (2006) 126(5):855–67. doi: 10.1016/j.cell.2006.08.019 16959566

[99] Kim HB Lee SW Mun CH Yoon JY Pai J Shin I . O-Linked n-acetylglucosamine glycosylation of p65 aggravated the inflammation in both fibroblast-like synoviocytes stimulated by tumor necrosis factor-alpha and mice with collagen induced arthritis. Arthritis Res Ther (2015) 17:248. doi: 10.1186/s13075-015-0762-7 26370562 PMC4570085

[100] Wolfert MA Boons GJ . Adaptive immune activation: Glycosylation does matter. Nat Chem Biol (2013) 9(12):776–84. doi: 10.1038/nchembio.1403 PMC3966069 24231619

[101] Li YN Chen CW Trinh-Minh T Zhu H Matei AE Gyorfi AH . Dynamic changes in O-GlcNAcylation regulate osteoclast differentiation and bone loss *via* nucleoporin 153. Bone Res (2022) 10(1):51. doi: 10.1038/s41413-022-00218-9 35879285 PMC9314416

[102] Kim MJ Kim HS Lee S Min KY Choi WS You JS . Hexosamine biosynthetic pathway-derived O-GlcNAcylation is critical for RANKL-mediated osteoclast differentiation. Int J Mol Sci (2021) 22(16). doi: 10.3390/ijms22168888 PMC8396330 34445596

[103] Takeuchi T Sugimoto A Imazato N . Glucosamine suppresses osteoclast differentiation through the modulation of glycosylation including O-GlcNAcylation. Biol Pharm Bull (2017) 40(3):352–6. doi: 10.1248/bpb.b16-00877 28250278

[104] Arnold JN Wormald MR Sim RB Rudd PM Dwek RA . The impact of glycosylation on the biological function and structure of human immunoglobulins. Annu Rev Immunol (2007) 25:21–50. doi: 10.1146/annurev.immunol.25.022106.141702 17029568

[105] Scherer HU van der Woude D Ioan-Facsinay A . Glycan profiling of anti-citrullinated protein antibodies isolated from human serum and synovial fluid. Arthritis Rheum (2010) 62(6):1620–9. doi: 10.1002/art.27414 20178128

[106] Andersson A Bernardi AI Nurkkala-Karlsson M Stubelius A Grahnemo L Ohlsson C . Suppression of experimental arthritis and associated bone loss by a tissue-selective estrogen complex. Endocrinology (2016) 157(3):1013–20. doi: 10.1210/en.2015-1820 26745543

[107] Rietjens IM Sotoca AM Vervoort J Louisse J . Mechanisms underlying the dualistic mode of action of major soy isoflavones in relation to cell proliferation and cancer risks. Mol Nutr Food Res (2013) 57(1):100–13. doi: 10.1002/mnfr.201200439 23175102

[108] Garcia PV Robinson LJ Borysenko CW Lehmann T Kalla SE Blair HC . Negative regulation of RANKL-induced osteoclastic differentiation in RAW264.7 cells by estrogen and phytoestrogens. J Biol Chem (2005) 280(14):13720–7. doi: 10.1074/jbc.M410995200 15644335

[109] Du N Song L Li Y Wang T Fang Q Ou J . Phytoestrogens protect joints in collagen induced arthritis by increasing IgG glycosylation and reducing osteoclast activation. Int Immunopharmacol (2020) 83:106387. doi: 10.1016/j.intimp.2020.106387 32172207

[110] Guo YC Zhang SW Yuan Q . Deubiquitinating enzymes and bone remodeling. Stem Cells Int (2018) 2018:3712083. doi: 10.1155/2018/3712083 30123285 PMC6079350

[111] Li C Qiu M Chang L Qi J Zhang L Ryffel B . The osteoprotective role of USP26 in coordinating bone formation and resorption. Cell Death Differ (2022) 29(6):1123–36. doi: 10.1038/s41418-021-00904-x PMC9177963 35091692

[112] Li Q Wang M Xue H Liu W Guo Y Xu R . Ubiquitin-specific protease 34 inhibits osteoclast differentiation by regulating NF-kappaB signaling. J Bone Miner Res (2020) 35(8):1597–608. doi: 10.1002/jbmr.4015 32212276

[113] Yamanaka S Sato Y Oikawa D Goto E Fukai S Tokunaga F . Subquinocin, a small molecule inhibitor of CYLD and USP-family deubiquitinating enzymes, promotes NF-kappaB signaling. Biochem Biophys Res Commun (2020) 524(1):1–7. doi: 10.1016/j.bbrc.2019.12.049 31898971

[114] Shalev M Arman E Stein M Cohen-Sharir Y Brumfeld V Kapishnikov S . PTPRJ promotes osteoclast maturation and activity by inhibiting cbl-mediated ubiquitination of NFATc1 in late osteoclastogenesis. FEBS J (2021) 288(15):4702–23. doi: 10.1111/febs.15778 33605542

[115] Chiou WF Huang YL Liu YW . (+)-vitisin a inhibits osteoclast differentiation by preventing TRAF6 ubiquitination and TRAF6-TAK1 formation to suppress NFATc1 activation. PloS One (2014) 9(2):e89159. doi: 10.1371/journal.pone.0089159 24558484 PMC3928435

[116] Meng J Zhang W Wang C Zhang W Zhou C Jiang G . Catalpol suppresses osteoclastogenesis and attenuates osteoclast-derived bone resorption by modulating PTEN activity. Biochem Pharmacol (2020) 171:113715. doi: 10.1016/j.bcp.2019.113715 31751538

[117] Li X Islam S Xiong M Nsumu NN Lee MW Zhang LQ . Epigenetic regulation of NfatC1 transcription and osteoclastogenesis by nicotinamide phosphoribosyl transferase in the pathogenesis of arthritis. Cell Death Discov (2019) 5:62. doi: 10.1038/s41420-018-0134-6 30774990 PMC6365567

[118] Asagiri M Sato K Usami T Ochi S Nishina H Yoshida H . Autoamplification of NFATc1 expression determines its essential role in bone homeostasis. J Exp Med (2005) 202(9):1261–9. doi: 10.1084/jem.20051150 PMC2213228 16275763

[119] Izawa N Kurotaki D Nomura S Fujita T Omata Y Yasui T . Cooperation of PU.1 with IRF8 and NFATc1 defines chromatin landscapes during RANKL-induced osteoclastogenesis. J Bone Miner Res (2019) 34(6):1143–54. doi: 10.1002/jbmr.3689 30721543

[120] Carey HA Hildreth BR Samuvel DJ Thies KA Rosol TJ Toribio RE . Eomes partners with PU.1 and MITF to regulate transcription factors critical for osteoclast differentiation. iScience (2019) 11:238–45. doi: 10.1016/j.isci.2018.12.018 PMC6327072 30634169

[121] Lu SY Li M Lin YL . Mitf induction by RANKL is critical for osteoclastogenesis. Mol Biol Cell (2010) 21(10):1763–71. doi: 10.1091/mbc.e09-07-0584 PMC2869381 20357005

[122] Lu SY Li M Lin YL . Mitf regulates osteoclastogenesis by modulating NFATc1 activity. Exp Cell Res (2014) 328(1):32–43. doi: 10.1016/j.yexcr.2014.08.018 25152440 PMC4177974

[123] Berridge MJ Lipp P Bootman MD . The versatility and universality of calcium signalling. Nat Rev Mol Cell Biol (2000) 1(1):11–21. doi: 10.1038/35036035 11413485

[124] Sato K Suematsu A Nakashima T Takemoto-Kimura S Aoki K Morishita Y . Regulation of osteoclast differentiation and function by the CaMK-CREB pathway. Nat Med (2006) 12(12):1410–6. doi: 10.1038/nm1515 17128269

[125] Lu DZ Dong W Feng XJ Chen H Liu J Wang H . CaMKII(delta) regulates osteoclastogenesis through ERK, JNK, and p38 MAPKs and CREB signalling pathway. Mol Cell Endocrinol (2020) 508:110791. doi: 10.1016/j.mce.2020.110791 32173349

[126] Park KH Gu DR Jin SH Yoon CS Ko W Kim YC . Pueraria lobate inhibits RANKL-mediated osteoclastogenesis *via* downregulation of CREB/PGC1beta/c-Fos/NFATc1 signaling. Am J Chin Med (2017) 45(8):1725–44. doi: 10.1142/S0192415X17500938 29121799

[127] Wang L Han L Xue P Hu X Wong SW Deng M . Dopamine suppresses osteoclast differentiation *via* cAMP/PKA/CREB pathway. Cell Signal (2021) 78:109847. doi: 10.1016/j.cellsig.2020.109847 33242564 PMC8691485

[128] Weivoda MM Ruan M Hachfeld CM Pederson L Howe A Davey RA . Wnt signaling inhibits osteoclast differentiation by activating canonical and noncanonical cAMP/PKA pathways. J Bone Miner Res (2016) 31(1):65–75. doi: 10.1002/jbmr.2599 26189772 PMC4758681

[129] Yoon SH Ryu J Lee Y Lee ZH Kim HH . Adenylate cyclase and calmodulin-dependent kinase have opposite effects on osteoclastogenesis by regulating the PKA-NFATc1 pathway. J Bone Miner Res (2011) 26(6):1217–29. doi: 10.1002/jbmr.310 21611964

[130] Tondravi MM McKercher SR Anderson K Erdmann JM Quiroz M Maki R . Osteopetrosis in mice lacking haematopoietic transcription factor PU. 1. Nat (1997) 386(6620):81–4. doi: 10.1038/386081a0 9052784

[131] Zhang LQ Van Haandel L Xiong M Huang P Heruth DP Bi C . Metabolic and molecular insights into an essential role of nicotinamide phosphoribosyltransferase. Cell Death Dis (2017) 8(3):e2705. doi: 10.1038/cddis.2017.132 28333140 PMC5386535

[132] Zhang LQ Heruth DP Ye SQ . Nicotinamide phosphoribosyltransferase in human diseases. J Bioanal BioMed (2011) 3:13–25. doi: 10.4172/1948-593X.1000038 22140607 PMC3227030

[133] Busso N Karababa M Nobile M Rolaz A Van Gool F Galli M . Pharmacological inhibition of nicotinamide phosphoribosyltransferase/visfatin enzymatic activity identifies a new inflammatory pathway linked to NAD. PloS One (2008) 3(5):e2267. doi: 10.1371/journal.pone.0002267 18493620 PMC2377336

[134] Presumey J Courties G Louis-Plence P Escriou V Scherman D Pers YM . Nicotinamide phosphoribosyltransferase/visfatin expression by inflammatory monocytes mediates arthritis pathogenesis. Ann Rheum Dis (2013) 72(10):1717–24. doi: 10.1136/annrheumdis-2012-202403 23313810

[135] Frye RA . Phylogenetic classification of prokaryotic and eukaryotic Sir2-like proteins. Biochem Biophys Res Commun (2000) 273(2):793–8. doi: 10.1006/bbrc.2000.3000 10873683

[136] Yeung F Hoberg JE Ramsey CS Keller MD Jones DR Frye RA . Modulation of NF-kappaB-dependent transcription and cell survival by the SIRT1 deacetylase. EMBO J (2004) 23(12):2369–80. doi: 10.1038/sj.emboj.7600244 PMC423286 15152190

[137] Shakibaei M Buhrmann C Mobasheri A . Resveratrol-mediated SIRT-1 interactions with p300 modulate receptor activator of NF-kappaB ligand (RANKL) activation of NF-kappaB signaling and inhibit osteoclastogenesis in bone-derived cells. J Biol Chem (2011) 286(13):11492–505. doi: 10.1074/jbc.M110.198713 PMC3064204 21239502

[138] Edwards JR Perrien DS Fleming N Nyman JS Ono K Connelly L . Silent information regulator (Sir)T1 inhibits NF-kappaB signaling to maintain normal skeletal remodeling. J Bone Miner Res (2013) 28(4):960–9. doi: 10.1002/jbmr.1824 23172686

[139] Hah YS Cheon YH Lim HS Cho HY Park BH Ka SO . Myeloid deletion of SIRT1 aggravates serum transfer arthritis in mice *via* nuclear factor-kappaB activation. PloS One (2014) 9(2):e87733. doi: 10.1371/journal.pone.0087733 24498364 PMC3912001

[140] Chiplunkar AR Lung TK Alhashem Y Koppenhaver BA Salloum FN Kukreja RC . Kruppel-like factor 2 is required for normal mouse cardiac development. PloS One (2013) 8(2):e54891. doi: 10.1371/journal.pone.0054891 23457456 PMC3573061

[141] Kuo CT Veselits ML Barton KP Lu MM Clendenin C Leiden JM . The LKLF transcription factor is required for normal tunica media formation and blood vessel stabilization during murine embryogenesis. Genes Dev (1997) 11(22):2996–3006. doi: 10.1101/gad.11.22.2996 9367982 PMC316695

[142] Parmar KM Nambudiri V Dai G Larman HB Gimbrone MA García-Cardeña G . Statins exert endothelial atheroprotective effects *via* the KLF2 transcription factor. J Biol Chem (2005) 280(29):26714–9. doi: 10.1074/jbc.C500144200 15878865

[143] Deng WG Zhu Y Wu KK . Role of p300 and PCAF in regulating cyclooxygenase-2 promoter activation by inflammatory mediators. Blood (2004) 103(6):2135–42. doi: 10.1182/blood-2003-09-3131 14630807

[144] Das H Kumar A Lin Z Patino WD Hwang PM Feinberg MW . Kruppel-like factor 2 (KLF2) regulates proinflammatory activation of monocytes. Proc Natl Acad Sci USA (2006) 103(17):6653–8. doi: 10.1073/pnas.0508235103 PMC1458936 16617118

[145] Das M Deb M Laha D Joseph M Kanji S Aggarwal R . Myeloid kruppel-like factor 2 critically regulates K/BxN serum-induced arthritis. Cells (2019) 8(8). doi: 10.3390/cells8080908 PMC6721677 31426355

[146] Rolph D Das H . Transcriptional regulation of osteoclastogenesis: The emerging role of KLF2. Front Immunol (2020) 11:937. doi: 10.3389/fimmu.2020.00937 32477372 PMC7237574

[147] Das M Lu J Joseph M Aggarwal R Kanji S McMichael BK . Kruppel-like factor 2 (KLF2) regulates monocyte differentiation and functions in mBSA and IL-1beta-induced arthritis. Curr Mol Med (2012) 12(2):113–25. doi: 10.2174/156652412798889090 PMC3286012 22280353

[148] Das M Laha D Kanji S Joseph M Aggarwal R Iwenofu OH . Induction of kruppel-like factor 2 reduces K/BxN serum-induced arthritis. J Cell Mol Med (2019) 23(2):1386–95. doi: 10.1111/jcmm.14041 PMC6349180 30506878

[149] Yamakawa T Okamatsu N Ishikawa K Kiyohara S Handa K Hayashi E . Novel gene merlot inhibits differentiation and promotes apoptosis of osteoclasts. Bone (2020) 138:115494. doi: 10.1016/j.bone.2020.115494 32569872

[150] Kewley RJ Whitelaw ML Chapman-Smith A . The mammalian basic helix-loop-helix/PAS family of transcriptional regulators. Int J Biochem Cell Biol (2004) 36(2):189–204. doi: 10.1016/S1357-2725(03)00211-5 14643885

[151] Ilvesaro J Pohjanvirta R Tuomisto J Viluksela M Tuukkanen J . Bone resorption by aryl hydrocarbon receptor-expressing osteoclasts is not disturbed by TCDD in short-term cultures. Life Sci (2005) 77(12):1351–66. doi: 10.1016/j.lfs.2005.01.027 15913656

[152] Iqbal J Sun L Cao J Yuen T Lu P Bab I . Smoke carcinogens cause bone loss through the aryl hydrocarbon receptor and induction of Cyp1 enzymes. Proc Natl Acad Sci USA (2013) 110(27):11115–20. doi: 10.1073/pnas.1220919110 PMC3704019 23776235

[153] Voronov I Heersche JN Casper RF Tenenbaum HC Manolson MF . Inhibition of osteoclast differentiation by polycyclic aryl hydrocarbons is dependent on cell density and RANKL concentration. Biochem Pharmacol (2005) 70(2):300–7. doi: 10.1016/j.bcp.2005.04.028 15919055

[154] Park R Madhavaram S Ji JD . The role of aryl-hydrocarbon receptor (AhR) in osteoclast differentiation and function. Cells (2020) 9(10). doi: 10.3390/cells9102294 PMC7602422 33066667

[155] Voronov I Li K Tenenbaum HC Manolson MF . Benzo[a]pyrene inhibits osteoclastogenesis by affecting RANKL-induced activation of NF-kappaB. Biochem Pharmacol (2008) 75(10):2034–44. doi: 10.1016/j.bcp.2008.02.025 18396263

[156] Ovrevik J Lag M Lecureur V Gilot D Lagadic-Gossmann D Refsnes M . AhR and arnt differentially regulate NF-kappaB signaling and chemokine responses in human bronchial epithelial cells. Cell Commun Signal (2014) 12:48. doi: 10.1186/s12964-014-0048-8 25201625 PMC4222560

[157] Schneider AJ Branam AM Peterson RE . Intersection of AHR and wnt signaling in development, health, and disease. Int J Mol Sci (2014) 15(10):17852–85. doi: 10.3390/ijms151017852 PMC4227194 25286307

[158] Wang Q Kurita H Carreira V Ko CI Fan Y Zhang X . Ah receptor activation by dioxin disrupts activin, BMP, and WNT signals during the early differentiation of mouse embryonic stem cells and inhibits cardiomyocyte functions. Toxicol Sci (2016) 149(2):346–57. doi: 10.1093/toxsci/kfv246 PMC4900218 26572662

[159] Occhi G Barollo S Regazzo D Bertazza L Galuppini F Guzzardo Vc . A constitutive active MAPK/ERK pathway due to BRAFV600E positively regulates AHR pathway in PTC. Oncotarget (2015) 6(31):32104–14. doi: 10.18632/oncotarget.5194 PMC4741662 26392334

[160] Donate PB Alves DLK Peres RS Almeida F Fukada SY Silva TA . Cigarette smoke induces miR-132 in Th17 cells that enhance osteoclastogenesis in inflammatory arthritis. Proc Natl Acad Sci USA (2021) 118(1). doi: 10.1073/pnas.2017120118 PMC7817209 33443169

[161] Smart SK Vasileiadi E Wang X . The emerging role of TYRO3 as a therapeutic target in cancer. Cancers (Basel) (2018) 10(12). doi: 10.3390/cancers10120474 PMC6316664 30501104

[162] Pagani S Bellan M Mauro D Castello LM Avanzi GC Lewis MJ . New insights into the role of Tyro3, axl, and mer receptors in rheumatoid arthritis. Dis Markers (2020) 2020:1614627. doi: 10.1155/2020/1614627 32051695 PMC6995487

[163] Rothlin CV Lemke G . TAM receptor signaling and autoimmune disease. Curr Opin Immunol (2010) 22(6):740–6. doi: 10.1016/j.coi.2010.10.001 PMC2997887 21030229

[164] Vomero M Barbati C Colasanti T Perricone C Novelli L Ceccarelli F . Autophagy and rheumatoid arthritis: Current knowledges and future perspectives. Front Immunol (2018) 9:1577. doi: 10.3389/fimmu.2018.01577 30072986 PMC6058034

[165] Henry DH Costa L Goldwasser F Hirsh V Hungria V Prausova J . Randomized, double-blind study of denosumab versus zoledronic acid in the treatment of bone metastases in patients with advanced cancer (excluding breast and prostate cancer) or multiple myeloma. J Clin Oncol (2011) 29(9):1125–32. doi: 10.1200/JCO.2010.31.3304 21343556

[166] Deodhar A Dore RK Mandel D Schechtman J Shergy W Trapp R . Denosumab-mediated increase in hand bone mineral density associated with decreased progression of bone erosion in rheumatoid arthritis patients. Arthritis Care Res (Hoboken) (2010) 62(4):569–74. doi: 10.1002/acr.20004 20391513

[167] Cohen SB Dore RK Lane NE Ory PA Peterfy CG Sharp JT . Denosumab treatment effects on structural damage, bone mineral density, and bone turnover in rheumatoid arthritis: A twelve-month, multicenter, randomized, double-blind, placebo-controlled, phase II clinical trial. Arthritis Rheum (2008) 58(5):1299–309. doi: 10.1002/art.23417 18438830

[168] Takeuchi T Tanaka Y Soen S Yamanaka H Yoneda T Tanaka S . Effects of the anti-RANKL antibody denosumab on joint structural damage in patients with rheumatoid arthritis treated with conventional synthetic disease-modifying antirheumatic drugs (DESIRABLE study): A randomised, double-blind, placebo-controlled phase 3 trial. Ann Rheum Dis (2019) 78(7):899–907. doi: 10.1136/annrheumdis-2018-214827 31036625 PMC6585575

[169] Perpetuo IP Caetano-Lopes J Rodrigues AM Campanilho-Marques R Ponte C Canhão H . Effect of tumor necrosis factor inhibitor therapy on osteoclasts precursors in rheumatoid arthritis. BioMed Res Int (2017) 2017:2690402. doi: 10.1155/2017/2690402 28286757 PMC5327780

[170] Guo Y Zhang X Qin M Wang X . Changes in peripheral CD19(+)Foxp3(+) and CD19(+)TGFbeta(+) regulatory b cell populations in rheumatoid arthritis patients with interstitial lung disease. J Thorac Dis (2015) 7(3):471–7. doi: 10.3978/j.issn.2072-1439.2015.02.11 PMC4387448 25922727

[171] Al-Bogami M Bystrom J Clanchy F Taher TE Mangat P Williams RO . TNFalpha inhibitors reduce bone loss in rheumatoid arthritis independent of clinical response by reducing osteoclast precursors and IL-20. Rheumatol (Oxford) (2021) 60(2):947–57. doi: 10.1093/rheumatology/keaa551 32984900

[172] Pettit AR Ji H von Stechow D Müller R Goldring SR Choi Y . TRANCE/RANKL knockout mice are protected from bone erosion in a serum transfer model of arthritis. Am J Pathol (2001) 159(5):1689–99. doi: 10.1016/S0002-9440(10)63016-7 PMC1867076 11696430

[173] Redlich K Hayer S Ricci R David JP Tohidast-Akrad M Kollias G . Osteoclasts are essential for TNF-alpha-mediated joint destruction. J Clin Invest (2002) 110(10):1419–27. doi: 10.1172/JCI0215582 PMC151809 12438440

[174] Moura RA Canhao H Polido-Pereira J Rodrigues AM Navalho M Mourao AF . BAFF and TACI gene expression are increased in patients with untreated very early rheumatoid arthritis. J Rheumatol (2013) 40(8):1293–302. doi: 10.3899/jrheum.121110 23772083

[175] van de Laar L Saelens W De Prijck S Martens L Scott CL Van Isterdael G . Yolk sac macrophages, fetal liver, and adult monocytes can colonize an empty niche and develop into functional tissue-resident macrophages. Immunity (2016) 44(4):755–68. doi: 10.1016/j.immuni.2016.02.017 26992565

